# Cultural Differences in People’s Psychological Response to COVID-19

**DOI:** 10.3389/fpsyg.2021.636062

**Published:** 2021-07-12

**Authors:** Suhui Yap, Albert Lee, Li-Jun Ji, Ye Li, Ying Dong

**Affiliations:** ^1^Department of Psychology, Queen’s University, Kingston, ON, Canada; ^2^Department of Psychology, School of Social Sciences, Nanyang Technological University, Singapore, Singapore; ^3^Department of Psychology, Central China Normal University, Wuhan, China; ^4^Faculty of Education, Central China Normal University, Wuhan, China

**Keywords:** COVID-19, state well - being, meaning in life, optimism, culture

## Abstract

The present research studied Chinese and Euro-Canadian students during the COVID-19 pandemic, focusing on their affect, optimism, well-being, and meaning in life. The results revealed both differences and similarities across cultures. As predicted, Chinese participants reported more positive affect and less negative affect, higher optimism, higher state psychological well-being, and higher meaning presence, compared to Euro-Canadian participants. The findings were replicated after a week’s delay. Analyses on longitudinal data showed that state optimism, state well-being, and meaning presence influenced one another over time. These variables also mediated the cultural differences in one another. These results are consistent with cultural work on naïve dialecticism and non-linear lay theory of change. Results also demonstrate underlying relationships among the constructs that are common to both cultural groups. Broadly, the present research highlights the impact of culture on people’s response to challenging life situations and the mechanisms underlying these cultural differences.

## Cultural Differences in People’s Psychological Response to COVID-19

Since December 2019, a new form of coronavirus has turned the world upside down. The pandemic has not only caused mass quarantines and deaths, but also generated high levels of stress due to fear of the virus and physical/social isolation. In Asia, divorce rates have skyrocketed unexpectedly, allegedly due to couples becoming fed up with each other from experiencing extended self-quarantine (Prasso, 2020; Sun, 2020). Travel bans, panic buying, and the closure of public spaces have all contributed to giving the world a harmful upsurge of stress and negativity, making the pandemic’s effects worse than they already are.

Despite all the difficulties and challenges presented by the pandemic, people are trying to react positively. Some people have taken the pandemic as an opportunity to bond with their families and friends, while others are reacting positively by offering help to their communities. These observations are in line with various ways of constructive coping, such as reacting positively to significant stressors (Dyer and McGuinness, 1996; Sinclair and Wallston, 2004), imbuing the future with a positive outlook (Scheier and Carver, 1985; Dember et al., 1989), and extracting positive meanings from bad experiences (Masten et al., 1990; O’Leary and Ickovics, 1995). The present research examines people’s psychological responses during the pandemic from a cross-cultural perspective, comparing Chinese and Euro-Canadian students. This research question was motivated by prior work, which revealed the role of culture in the way people think about and understand the world (e.g., Peng and Nisbett, 1999; Ji et al., 2001; Lee et al., 2020), with implications for how they respond to highly aversive events. Applying such cultural differences to the context of COVID-19 suggests that compared to Euro-Canadians, Chinese may be more inclined to react to the pandemic with positivity in terms of optimism, well-being, and meaning in life, compared to Euro-Canadians. We discuss these constructs in turn.

### Optimism and Culture

Under the threat of a life-changing event, such as a pandemic, how do people brighten the seemingly dim future? What mental processes are in operation when people manage to keep their hopes high? These questions are related to optimism, which is typically known as the positive expectations people have about the future (Scheier and Carver, 1985; Dember et al., 1989). In the literature, optimism has been conceptualized and measured as a stable trait assumed to change little over time (e.g., Scheier and Carver, 1992, 2018). This assumption, however, has been challenged by recent research with a state view of optimism, which assumes that people’s expectations about the future are fluid, subject to environmental cues, context-specific, and thus malleable in response to salient reminders (Millstein et al., 2019). In this view, state optimism may fluctuate in the context of the COVID-19 pandemic.

As a psychological construct, optimism has been shown to play a key role in well-being. For example, people who are optimistic are less likely to use alcohol (Wray et al., 2013) or drugs (Carvajal et al., 1998) than people who are not optimistic. When times are tough, optimism has been shown to predict a host of favorable emotional and behavioral outcomes, including problem-focused coping (Friedman et al., 1992; Fournier et al., 2002), positive reappraisal (Slattery et al., 2017), active goal pursuit (e.g., Carver et al., 1983), and health-enhancing behaviors (Robbins et al., 1991). On the flipside, optimism is negatively associated with distress emotions (Aspinwall and Taylor, 1992), PTSD symptoms (Frazier et al., 2011) and suicidal behaviors (Fawcett et al., 1987).

In principle, optimism has to do with positive anticipations when things go wrong. That is, people who are optimistic tend to have the anticipation that a situation, no matter how dark it feels at the moment, will eventually be replaced by a brighter outcome. Such a view is highly congruent with the non-linear theory of change (Ji et al., 2001; Ji, 2008) common in East Asian cultures, which assumes that everything in the world is in constant transformation between negativity and positivity, like the waxing and waning of the moon. Cultural differences in optimism are also compatible with naïve dialecticism (Peng and Nisbett, 1999), a mental pillar of reasoning in East Asian cultures which assumes that contradictions are the foundation of life. Through the window of naïve dialecticism, life events, no matter how bad, would always contain the seed of good, and vice versa. Both the non-linear theory of change and naïve dialecticism have been shown to be stronger among Chinese than among North Americans (see Spencer-Rodgers et al., 2010a for a review).

In line with this claim, past work has shown that non-linear thinking Chinese participants were more likely to appraise the SARS outbreak with optimism and positive thinking—for example, they reported more positive changes to their lives—compared to Euro-Canadian participants who assumed linearity in change (Ji et al., 2004). Furthermore, cultural differences in optimism may have to do with the fact that suffering in life is construed more positively by Chinese than by Euro-Canadians (Ji et al., 2020), corresponding to the themes of Buddhism and Taoism in East Asian cultures (Ji et al., 2010). Furthermore, Ji et al. (2021) have shown that Chinese participants were more optimistic than Euro-Canadians in response to negative events, traceable to cultural differences in the non-linear theory of change.

In sum, optimism is associated with a wide range of mental, emotional, and behavioral outcomes, many of which are beneficial for people who are going through life adversities. Drawing from past research, we hypothesized that in the context of the COVID-19 pandemic, Chinese participants would feel more optimistic than would Euro-Canadian participants (Hypothesis 1). Optimism aside, would culture play a role in other responses to life adversities, such as the recognition of meaning in life and psychological well-being?

### Meaning in Life and Culture

When people are faced with adversities, finding meaning in life can contribute to optimism. At a broad level, meaning in life has been discussed under the grand theme of purposefulness (e.g., Battista and Almond, 1973; Klinger, 1977). At a more concrete level, meaning is manifested in the awareness of purposes or significance (Ryff and Singer, 1998) from the environment that are otherwise hidden from view. For example, a person bound to a wheelchair may discover the presence of meaning by turning to art. Likewise, instead of dwelling on the bad side of the COVID-19 pandemic, some people may come to recognize new meaning in life through new hobbies, unexplored career paths, tighter bonds with loved ones, or the appreciation of nature.

The recognition of meaning often involves the discovery of new perspectives. Life adversities may feel less painful when a person manages to take a step back from the problems at hand, understand them in a broader context, and integrate new perspectives with old ones. Deriving meaning through different perspectives is a key ingredient of wisdom, according to empirical scientists (e.g., Baltes and Staudinger, 2000; Grossmann et al., 2013) as well as philosophers. In particular, Taoism and Buddhism are known for their emphasis on perspectives as a source of wisdom (Yamamoto, 1998; Birren and Svensson, 2005). Knowledge makes people smart, but to be wise, one must go beyond the surface of things, attend to a broader context for underlying trends, and generate new interpretations from multiple perspectives, even if they contradict one another. The acquisition of new meaning through contexts, trends, and perspectives is consistent with naïve dialecticism and the non-linear theory of change – both of which are more prevalent in East Asian than North American cultures (e.g., Peng and Nisbett, 1999; Ji et al., 2001; Ji, 2008). This claim is supported by empirical work. For example, studies have shown that compared to North Americans, East Asians tend to have a greater focus on contextual information (Morris and Peng, 1994; Ji et al., 2000), a wider mental frame for temporal patterns and trends (Ji et al., 2009, 2019), and a stronger tendency for perspective-taking (e.g., Cohen et al., 2007; Wu and Keysar, 2007). The awareness for unapparent information, such as seeing positive meaning in a negative situation (e.g., fights between a couple can get them to know each other better) or finding insight in the mundane (e.g., the awe of nature through gardening), is also more common in East Asian than North American cultures (Ji et al., 2004, 2020; Grossmann et al., 2014, 2016). All these findings seem to suggest cultural differences in finding meaning when life is troubled by adversities.

Research has shown positive connections between trait optimism and meaning in life. For example, optimism has been shown to predict meaning in life (Kealy et al., 2020). Trait optimism facilitates subjective well-being and good health (Wrosch and Scheier, 2003). Likewise, finding meaning in life positively predicts optimism, which in turn increases psychological well-being (Ho et al., 2010). The association between meaning in life and optimism is found in Asian Americans and European Americans (Yu and Chang, 2019), and in young and older adults (Krause, 2003). Thus, the relationship between optimism (as a trait, at least) and meaning in life seems to be bidirectional, although most of these findings were based on cross-sectional research and trait measures of optimism.

Optimism is also consistently associated with psychological well-being. In a longitudinal study, Daukantaite and Bergman (2005) examined participants’ optimism at age 13 and their subjective well-being at age 43. Results showed that measures of optimism at an early age consistently predicted subjective well-being at later stages in life, suggesting that the role of optimism in well-being is robust and stable over time. Numerous other studies have revealed similar patterns (e.g., Scheier and Carver, 1992; Halama and Dedova, 2007; Ho et al., 2010), demonstrating associations between optimism and a host of benefits, including subjective well-being (Aglozo et al., 2019) and conceptually related constructs such as positive affect (Carver et al., 2010) and life satisfaction (Duffy et al., 2013). Well-being and its relationship with culture will be discussed in greater details below.

In sum, the tendency to view the future in a positive light may promote people’s well-being and a sense of meaning in their lives as the COVID-19 pandemic looms over the world. Success in finding meaning often stems from the tendency to examine an existing event from new perspectives. Such tendency may be more apparent among East Asians than among North Americans, as past findings suggest. Applying these findings to context, one may expect a stronger inclination to find meaning in the COVID-19 pandemic among Chinese participants than among Euro-Canadian participants (Hypothesis 2). Considering that optimism, meaning in life, and well-being are intertwined and that cultural differences are expected in optimistic response to the pandemic, one may also expect cultural differences in meaning and well-being.

### Psychological Well-Being and Culture

People who feel hopeful when things go wrong, appreciate the good sides in bad, or see new meaning when there seems to be none, should, in principle, enjoy greater psychological well-being than people who do not. What does the literature say about this? For decades, psychological well-being has been recognized as one of the most crucial constructs of social life, studied by psychologists in multiple fields. In one approach, typically known as the hedonic approach, well-being concerns the extent to which one feels happy or satisfied with life. This approach essentially involves the attainment of pleasure and avoidance of pain, manifested in positive and negative emotions (Ryan and Deci, 2001; Diener, 2009). In another approach, typically known as the eudaimonic approach, well-being concerns the ability to strive for true potentials and life achievements. This approach essentially involves personal growth through the pursuits of wisdom and virtues, and is closely related to finding meaning in life (Ryff and Keyes, 1995; Ryan and Deci, 2001). Both approaches emphasize different aspects of well-being and, together, they represent a good and fulfilling life that is worth living.

But what constitutes a good, fulfilling life? It depends on who is asking. For example, prior work has revealed cultural differences in the hedonic component of well-being, operationalized by the experience of positive affect. Results showed that while positive affect is generally appealing to everyone, culture does play a role in the kinds of positive affect people find as important or ideal for well-being. For example, North Americans are known for their emphasis on self-centered affect (e.g., pride, anger), or affect that concerns the attributes of the self, whereas East Asians tend to emphasize other-centered affect (e.g., respectful, shame), or affect that stems from significant others (Chow and Berenbaum, 2012). Hedonic differences in well-being are typically linked to the awareness for social contexts, more prevalent in East Asian than North American cultures (Nisbett, 2003). From other work, we also learn that affective experiences and well-being may have to do with the cultural assumptions people have about the world. For instance, unlike many North Americans, East Asians tend to perceive affective experiences and well-being as fluid, impermanent, and always changing, compatible with naïve dialecticism and a non-linear view of the world (Uchida and Kitayama, 2009). Similar observations have emerged from other studies (e.g., Miyamoto and Ma, 2011; see also Spencer-Rodgers et al., 2010a). These perspectives suggest that positive and negative affect need not be on the two ends of a continuum; the presence of one does not imply the absence of the other. Consequently, compared to North Americans whose tend to have polarized experience of affect (e.g., feeling happy with not sad), East Asians are more inclined to experience the co-occurance of opposing affect, such as feeling happy and unhappy at the same time (e.g., Bagozzi et al., 1999; Kitayama et al., 2000; Napa Scollon et al., 2005; Spencer-Rodgers et al., 2010b). This body of work is consistent with cultural differences in well-being, with East Asians more likely to perceive negativity as the seeds of positivity (e.g., Ji et al., 2001), respond to negative events with flexibility (Cheng, 2009), or, more generally, “finding the good in the bad” (Spencer-Rodgers et al., 2010a), relative to North Americans. Collectively, these findings forge links with other research showing that the thinking style of East Asians may dilute the negative impacts of stressful events and contribute to how pleasant, positive, or satisfied they feel about the stressful events (Ji et al., 2001; Hou et al., 2003).

Another common measure for psychological well-being focuses less on affective experiences per se and more broadly on the things that make a life good, satisfying, fulfilling, and worth living for. Is well-being nothing but a large bag of positivity? Or is it a delicate balance between happy and sad times? It depends. To many North Americans, life is satisfying when it is imbued by positive events. In contrast, to many East Asians, life is satisfying when it is a good mix of happy and sad times (Oishi, 2002). These findings resonate with other work (Ji et al., 2001), showing that Euro-Americans expected life happiness to follow a linear trend, whereas Chinese expected life happiness to change in a nonlinear way – to them, happiness and unhappiness transform to each other over time. Together, these findings highlight the impact of culture on well-being, in terms of what it constitutes and how it is manifested during challenging times.

To the extent that optimism is a predictor of well-being, Chinese participants should, by implication, report greater well-being in response to the pandemic, compared to Euro-Canadian participants (Hypothesis 2). In addition, ample research has revealed links between optimism, meaning in life, and well-being, conceptually and empirically (e.g., Carvajal et al., 1998; Wrosch and Scheier, 2003; Ho et al., 2010; Wray et al., 2013; Kealy et al., 2020). Drawing on these findings, one might expect state optimism to predict psychological well-being and meaning in life (Hypothesis 3). Finally, if optimism can be a source of well-being and meaning, and if optimism varies across cultures (e.g., Ji et al., 2004, 2020, 2021), then state optimism may mediate cultural differences in psychological well-being and meaning in life (Hypothesis 4).

### The Present Research

The present research aimed to unpack the connections among culture, optimism, psychological well-being, and meaning in life in the context of the COVID-19 pandemic. We conducted a cross-cultural study with over 500 Euro-Canadian and Chinese participants. In the study, all participants were instructed to complete the survey twice—about one week apart—but not everyone completed both surveys. The purpose of the longitudinal design is twofold: (1) to test the reliability of the findings, and (2) to investigate the relationships among variables across time and across cultures.

Prior cultural work has shown that East Asian and North American cultures are characterized by distinct assumptions about the world. Dialecticism and non-linearity are more central to the thinking styles of East Asians, such as Chinese, relative to the thinking styles of North Americans, such as Euro-Canadians (e.g., Peng and Nisbett, 1999; Ji et al., 2001). These findings suggest that Chinese should be more likely to see the silver lining of the pandemic, compared to Euro-Canadians. If so, Chinese participants would be more likely to respond to the pandemic in a positive way (i.e., more optimistic, better psychological well-being and higher meaning in life), relative to Euro-Canadian participants. No specific predictions were made about trait optimism given the mixed evidence in the literature^1^.

In summary, we aimed to examine the following predictions:

Hypothesis 1: Chinese participants should have higher state optimism than Euro-Canadian participants.

Hypothesis 2: Chinese participants would report better psychological well-being and higher meaning in life than Euro-Canadian participants.

In addition, given the empirical links between state optimism, psychological well-being, and meaning in life, we also examined the following predictions:

Hypothesis 3: State optimism may predict psychological well-being and meaning in life.

Hypothesis 4: State optimism may mediate cultural differences in psychological well-being and meaning in life.

While cross-sectional data can be used to examine these predictions, longitudinal data have the unique advantage of capturing whether, and to what extent, different variables may predict each other or mediate cultural differences over time. Thus, we will address the first two predictions with cross-sectional data and the last two predictions with longitudinal data.

## Method

### Participants

The present study was conducted in late March and early April 2020, a time when Canada and China were impacted by the pandemic. At Time 1, 293 Euro-Canadians (242 women, 50 men and one other; *M*_age_ = 20.66, *SD*_age_ = 3.72) from Queen’s University in Kingston, Canada, and 266 Chinese (220 women and 46 men; *M*_age_ = 19.88, *SD*_age_ = 1.07) students from the Central China Normal university in Wuhan, China^2^, completed the study. A week later at Time 2, 243 Euro-Canadians (205 women, 37 men, and 1 other) and 240 Chinese (201 women and 39 men) completed similar measures. Among the participants, 223 Euro-Canadians (188 women, 34 men, and 1 other) and 235 Chinese (196 women and 39 men) did the study at both times^3^.

All participants completed the study in their native language. The study material was first developed in English, and then translated into Chinese. Two bilingual researchers verified the translation to ensure its equivalence across language.

### Measures and Procedure

Participants completed the study online via Qualtrics. At each time, they reported their current affect, psychological well-being, optimism, and meaning in life. Due to time constraints, fewer measures were included at Time 2. Time 1 testing included the following measures^4^ :

(1) Current affect: Participants reported (0 = *not at all*, 9 = *very*) how *distressed, scared, anxious, worried, angry, depressed, nervous, hopeless, relaxed*, and *happy* they were feeling “*overall these days*” during the pandemic. Six of these items were selected from the affect measures used in Bruehlman-Senecal et al. (2016), in addition to four items we added (*distressed, hopeless, worries, relaxed*). These items were chosen because of their relevance to people’s responses to the pandemic. Current affect was measured so that we could gain a general picture of participants’ psychological state or a proxy of their well-being.
(2) Optimism: State optimism was measured with the State Optimism Measure (SOM-7; Millstein et al., 2019) and trait optimism with the revised Life Orientation Task (LOT-R; Scheier et al., 1994). The SOM-7 contains 7 items that captured participants’ tendency to feel positive about the future (e.g., “*At the moment, I expect more to go right than wrong when it comes to my future*”). The LOT-R includes 6 test items that capture participants’ general expectations about the future (e.g., “*In uncertain times I usually expect the best*”). For both scales, participants indicated (1 = *strongly disagree*, 5 = *strongly agree*) their endorsement of each item.
(3) Well-being: Participants completed two measures of well-being, one being the adapted WHO-Ten Well-Being index (Bech et al., 1996) and the other being the Satisfaction with Life Scale (SWLS; Diener et al., 1985). The adapted WHO-Ten index measured state-like well-being with 10 items, focusing on “*the absence of negative symptoms (i.e., anxiety, depression) and the presence of positive symptoms (e.g., energy)*” (Cooke et al., 2016, p. 743). Participants rated (0 = *not at all*, 7 = *very much*) how they felt *at the moment* (e.g., “*I feel happy, satisfied or pleased with my personal life*.”). The measure aligns with the hedonic component of well-being, centering around positive and negative feelings. SWLS measured the global perception of life with 5 items, with participants rating (1 = *strongly disagree*, 7 = *strongly agree*) the extent to which they were satisfied with their lives in general (e.g., “*In most ways my life is close to my ideal*”). In sum, the WHO index measured participants’ state well-being while SWLS measured their general perception of well-being.
(4) Meaning in life: Participants rated (1 = *absolutely untrue*, 7 = *absolutely true*) the extent to which they perceived meaning in life with the Meaning in Life Questionnaire (Steger et al., 2006). The scale includes 10 items, measuring the presence of meaning (e.g., “*My life has a clear sense of purpose*”) and search for meaning (e.g., “*I am seeking a purpose or mission for my life*”) respectively.

About a week later at Time 2, participants completed the same measures as at Time 1, except LOT-R.

## Results

We included all participants for cross-sectional analyses. For cross-time analyses, we included only participants who provided data at both time points.

As seen in Table 1, the zero-order correlations across cultural groups are consistent with prior work. For example, when all participants were analyzed regardless of culture, state optimism, well-being, and meaning in life were all positively correlated with one another.

**TABLE 1:** Correlation among main variables.

**Measures**	**1**	**2**	**3**	**4**	**5**	**6**	**7**
Euro-Canadians							
(1) State Optimism T1	—						
(2) State Well-being T1	0.76**	—					
(3) SWLS T1	0.65**	0.61**	—				
(4) Meaning Presence T1	0.55**	0.52**	0.45**	—			
(5) State Optimism T2	0.73**	0.59**	0.58**	0.47**	—		
(6) State Well-being T2	0.63**	0.67**	0.49**	0.41**	0.70**	—	
(7) SWLS T2	0.56**	0.54**	0.78**	0.45**	0.66**	0.67**	—
(8) Meaning Presence T2	0.52**	0.50**	0.47**	0.80**	0.54**	0.50**	0.51**
Chinese							
(1) State Optimism T1	—						
(2) State Well-being T1	0.57**	—					
(3) SWLS T1	0.47**	0.60**	—				
(4) Meaning Presence T1	0.50**	0.52**	0.39**	—			
(5) State Optimism T2	0.74**	0.50**	0.40**	0.46**	—		
(6) State Well-being T2	0.39**	0.56**	0.49**	0.43**	0.54**	—	
(7) SWLS T2	0.33**	0.46**	0.66**	0.36**	0.49**	0.65**	—
(8) Meaning Presence T2	0.42**	0.45**	0.38**	0.67**	0.54**	0.55**	0.53**

T1,Time 1; T2,Time 2; SWLS, Satisfaction with Life Scale. **p < 0.001.

Next, we examined cultural differences in current affect, optimism, psychological well-being, and meaning in life. Results at Time 2 fully replicated results at Time 1. Then we tested how the key variables of interest (e.g., optimism, psychological well-being, and meaning in life) were related to one another over time. Degrees of freedom varied due to occasional missing data.

We have conducted measurement invariance tests and established partial measurement scalar invariance for state optimism, state well-being, and meaning presence (see details in the Supplementary Material). Cross-cultural comparisons based on invariant items showed similar patterns of results as those based on full scale items. The latter are reported in the paper, while the former can be found in Supplementary Material.

### Cultural Differences in Each of the Variables

#### Culture and Current Affect

We averaged ratings of current affect into two composites, one for positive affect (α_CA_ = 0.70 and α_CH_ = 0.68) and the other for negative affect (α_CA_ = 0.93 and α_CH_ = 0.92). At Time 1, Chinese participants reported higher positive affect (*M* = 5.14, *SD* = 1.58) than Euro-Canadian participants (*M* = 4.60, *SD* = 1.61), *F*(1, 557) = 16.28, *p* < 0.001, $\eta_{p}^{2}$ = 0.03, and lower negative affect (*M* = 3.96, *SD* = 1.75) than Euro-Canadian participants (*M* = 4.43, *SD* = 1.96), *F*(1, 557) = 9.04, *p* = 0.003, $\eta_{p}^{2}$ = 0.02.

At Time 2, we averaged ratings of positive affect into one composite (α_CA_ = 0.69 and α_CH_ = 0.78), and negative affect into another (α_CA_ = 0.93 and α_CH_ = 0.94). Results showed that at Time 2, Chinese participants reported higher positive affect (*M* = 5.39, *SD* = 1.47) than Euro-Canadian participants (*M* = 4.87, *SD* = 1.50), *F*(1, 481) = 15.02, *p* < 0.001, $\eta_{p}^{2}$ = 0.03, and lower negative affect (*M* = 3.57, *SD* = 1.73) than Euro-Canadian participants (*M* = 4.13, *SD* = 1.90), *F*(1, 481) = 11.35, *p* = 0.001, $\eta_{p}^{2}$ = 0.02. These results are in line with the distinct assumptions people have about affective experiences (e.g., Miyamoto and Ma, 2011), their states of well-being in response to the pandemic, and their views about negative events in life (e.g., Ji et al., 2001; Oishi, 2002).

#### Culture and Optimism

Was optimism higher among Chinese than Euro-Canadian participants (Hypothesis 1)? State optimism was computed by averaging the ratings of all items on the State Optimism Measure at Time 1 (α_CA_ = 0.91 and α_CH_ = 0.87) and at Time 2 (α_CA_ = 0.91 and α_CH_ = 0.89), respectively.

As expected, Chinese participants reported higher state optimism (*M* = 3.51, *SD* = 0.70) than Euro-Canadian participants (*M* = 3.34, *SD* = 0.83) at Time 1, *F*(1, 556) = 6.70, *p* = 0.010, $\eta_{p}^{2}$ = 0.01. At Time 2, Chinese participants (*M* = 3.49, *SD* = 0.68) also scored higher than Euro-Canadian participants (*M* = 3.32, *SD* = 0.83), *F*(1, 481) = 6.00, *p* = 0.015, $\eta_{p}^{2}$ = 0.01.

Trait optimism was only measured at Time 1 using LOT-R (α_CA_ = 0.83 and α_CH_ = 0.62). The scale did not establish measure invariance across cultures, and thus no meaningful comparison could be made across the two culture groups (see specific results in the Supplementary Material).

#### Culture, Psychological Well-Being, and Meaning

Were psychological well-being and meaning in life higher among Chinese than Euro-Canadian participants (Hypothesis 2)? As a measure of state well-being, ratings of adapted WHO items were averaged at Time 1 (α_CA_ = 0.91 and α_CH_ = 0.86) and at Time 2 (α_CA_ = 0.91 and α_CH_ = 0.88), respectively.

Chinese (*M* = 3.48, *SD* = 0.94) scored higher than Euro-Canadians (*M* = 2.83, *SD* = 1.25) on state well-being at Time 1, *F*(1, 557) = 47.81, *p* < 0.001, $\eta_{p}^{2}$ = 0.08. These results were replicated at Time 2: Chinese (*M* = 4.30, *SD* = 0.97) scored higher than Euro-Canadians (*M* = 3.55, *SD* = 1.31), *F*(1, 481) = 50.69, *p* < 0.001, $\eta_{p}^{2}$ = 0.10.

As a measure of general life satisfaction, SWLS did not establish scalar invariance, thus no cultural comparison could be made on this variable. For the sake of completion, we ran the analyses and reported the results in the Supplementary Material.

The Meaning in Life Questionnaire includes two subscales: the presence of meaning (α_CA_ = 0.88 and α_CH_ = 0.84 at Time 1, and α_CA_ = 0.89 and α_CH_ = 0.84 at Time 2) and the search for meaning (α_CA_ = 0.89 and α_CH_ = 0.85 at Time 1, and α_CA_ = 0.92 and α_CH_ = 0.87 at Time 2).

At Time 1, Chinese (*M* = 4.75, *SD* = 0.94) reported higher presence of meaning than did Euro-Canadians (*M* = 4.32, *SD* = 1.30), *F*(1, 553) = 20.11, *p* < 0.001, $\eta_{p}^{2}$ 0.04. The two groups did not differ in meaning search (*M* = 5.17, *SD* = 0.82 for Chinese; *M* = 5.07, *SD* = 1.18 for Euro-Canadians), *F*(1, 553) = 1.49, *p* = 0.222. Controlling for participants’ current affect, cultural differences in meaning presence remained significant, *F*(1, 551) = 11.31, *p* = 0.001, $\eta_{p}^{2}$ = 0.02.

Time 2 showed the same pattern of results: Chinese (*M* = 4.69, *SD* = 0.93) reported higher presence of meaning than did Euro-Canadians (*M* = 4.34, *SD* = 1.24), *F*(1, 479) = 12.21, *p* = 0.001, $\eta_{p}^{2}$ = 0.03, while the two culture groups did not differ in their search for meaning (*M* = 5.09, *SD* = 0.84 for Chinese; *M* = 4.91, *SD* = 1.22 for Euro-Canadians), *F*(1, 479) = 3.52, *p* = 0.061. Also, cultural differences in meaning presence remained significant while controlling for current affect, *F*(1, 477) = 4.02, *p* = 0.046.

### Longitudinal Effects

Did state optimism predict psychological well-being and meaning in life (Hypothesis 3)? We examined the relationships across time. We investigated how variables at Time 1 may predict variables at Time 2, and how such relationships may vary across cultures. The cross-time analyses were done based on the data from participants who did the study at both times. We ran a series of regressions, in the following format:

y2 ∼ y1 + x1 + culture + x1^∗^culture

In the model, y2 was the outcome variable at Time 2; y1 was the same outcome variable measured at time 1 and served as a covariate; x1 was the predictor variable at Time 1, whose interaction effect with culture was x1^∗^culture. All continuous variables were centered. Culture was coded as Canada = -0.5 and China = +0.5. We reported only significant effects in the following analyses.

#### State Optimism at Time 1 Predicts State Well-Being at Time 2

Controlling for well-being at Time 1 (*b* = 0.51, *t* = 9.66, *p* < 0.001), state optimism at Time 1 positively predicted well-being at Time 2 (*b* = 0.28, *t* = 3.80, *p* < 0.001), and so did culture (*b* = 0.34, *t* = 3.93, *p* < 0.001). In addition, the interaction of culture and optimism was significant (*b* = -0.23, *t* = -2.05, *p* = 0.041). Simple slope tests showed that optimism positively predicted well-being for Euro-Canadians (*b* = 0.40, *t* = 4.35, *p* < 0.001), but the effect was weaker and only marginally significant for Chinese (*b* = 0.17, *t* = 1.83, *p* = 0.068).

#### State Optimism at Time 1 Predicts Meaning Presence at Time 2

Controlling for meaning presence at Time 1 (*b* = 0.66, *t* = 19.13, *p* < 0.001), state optimism at Time 1 positively predicted meaning presence at Time 2 (*b* = 0.16, *t* = 3.02, *p* = 0.003). No other effect was significant (*t*s < 1, *p*s > 0.350).

Although not part of our predictions, we also examined the other combinations of the relationships among the three variables (state optimism, state well-being and meaning presence) and report them below.

#### State Well-Being at Time 1 Predicts State Optimism at Time 2

Controlling for state optimism at Time 1 (*b* = 0.67, *t* = 15.47, *p* < 0.001), state well-being at Time 1 positively predicted state optimism at Time 2 (*b* = 0.06, *t* = 2.06, *p* = 0.040). No other effect was significant (*t*s < 1, *p*s > 0.520).

#### Meaning Presence at Time 1 Predicts State Optimism at Time 2

Controlling for state optimism at Time 1 (*b* = 0.68, *t* = 18.49, *p* < 0.001), meaning presence at Time 1 positively predicted state optimism at Time 2 (*b* = 0.07, *t* = 2.77, *p* = 0.006). No other effect was significant (*t*s < 1, *p*s > 0.450).

#### Meaning Presence at Time 1 Predicts State Well-Being at Time 2

Controlling for state well-being at Time 1 (*b* = 0.60, *t* = 13.58, *p* < 0.001), meaning presence at Time 1 positively predicted state well-being at Time 2 (*b* = 0.13, *t* = 2.82, *p* = 0.005), and so did culture (*b* = 0.28, *t* = 3.20, *p* = 0.002). The interaction of culture and meaning presence was not significant (*b* = 0.03, *t* = 0.38, *p* = 0.701).

#### State Well-Being at Time 1 Predicts Meaning Presence at Time 2

Controlling for meaning presence at Time 1 (*b* = 0.66, *t* = 19.39, *p* < 0.001), state well-being at Time 1 positively predicted meaning presence at Time 2 (*b* = 0.12, *t* = 3.28, *p* = 0.001). No other effect was significant (*t*s < 1.12, *p*s > 0.265).

Together, these results revealed bi-directional, temporal relationships among state optimism, well-being, and meaning presence. For example, state optimism at Time 1 predicted subsequent well-being and meaning presence at Time 2; meaning presence and well-being at Time 1 predicted subsequent state optimism at Time 2.

### Cross-Time Mediation Analyses

Digging into underlying pathways, did state optimism mediate cultural differences in psychological well-being and meaning in life (Hypothesis 4)? Given the longitudinal nature of our data, we conducted cross-time mediation analyses to investigate this. That is, we examined whether state optimism at Time 1 would mediate cultural differences in well-being or meaning presence at Time 2. We conducted the following mediation analyses using the lavaan package (Rosseel, 2012) in R (R Core Team, 2019). Culture (China = 0.5, Canada = -0.5) was the independent variable. The dependent variable was either state well-being or meaning presence at Time 2. The mediator was state optimism at Time 1. In each analysis, we also controlled for the same measure of the dependent variable at Time 1.

As seen in Table 2, based on joint-significance tests (Yzerbyt et al., 2018), state optimism at Time 1 mediated cultural differences in state well-being and meaning presence at Time 2, respectively. Consistent with the conclusion from the joint-significance tests, the 95% percentile bootstrap confidence intervals for both indirect effects did not contain 0.

**TABLE 2:** Cross-time mediation results.

**DV (Time 2)**	**Mediator (Time 1)**	**a path (Culture → mediator)**	**b path (Mediator → DV)**	**c’ path (IV → DV controlling for mediator)**	**ab (indirect effect)**	**95% CI for indirect effect**
Well-being	Optimism	*b* = 0.20,	*b* = 0.29,	*b* = 0.33,	*b* = 0.06,	[0.01, 0.12]
		*z* = 2.79,	*z* = 3.62,	*z* = 3.92,	*z* = 2.17,	
		*p* = 0.005	*p* < 0.001	*p* < 0.001	*p* = 0.030	
Meaning	Optimism	*b* = 0.20,	*b* = 0.16,	*b* = -0.03,	*b* = 0.03,	[0.01, 0.07]
		*z* = 2.70,	*z* = 2.99,	*z* = -0.41,	*z* = 2.03,	
		*p* = 0.007	*p* = 0.003	*p* = 0.682	*p* = 0.042	
Optimism	Well-being	*b* = 0.69,	*b* = 0.06,	*b* = -0.03,	*b* = 0.04,	[0.00, 0.09]
		*z* = 6.67,	*z* = 1.96,	*z* = -0.60,	*z* = 1.88,	
		*p* < 0.001	*p* = 0.051	*p* = 0.549	*p* = 0.060	
Optimism	Meaning	*b* = 0.48,	*b* = 0.07,	*b* = -0.02,	*b* = 0.03,	[0.01, 0.06]
		*z* = 4.52,	*z* = 2.50,	*z* = -0.37,	*z* = 2.22,	
		*p* < 0.001	*p* = 0.012	*p* = 0.715	*p* = 0.026	
Well-being	Meaning	*b* = 0.48,	*b* = 0.12,	*b* = 0.28,	*b* = 0.06,	[0.01, 0.11]
		*z* = 4.45,	*z* = 2.57,	*z* = 3.27,	*z* = 2.30,	
		*p* < 0.001	*p* = 0.010	*p* = 0.001	*p* = 0.022	
Meaning	Well-being	*b* = 0.69,	*b* = 0.12,	*b* = -0.08,	*b* = 0.09,	[0.03, 0.15]
		*z* = 6.73,	*z* = 3.32,	*z* = -1.16,	*z* = 2.22,	
		*p* < 0.001	*p* = 0.001	*p* = 0.246	*p* = 0.003	

*Optimism refers to state optimism. Well-being refers to state well-being. Meaning refers to meaning presence.*

Next, we conducted similar mediation analyses with state optimism at Time 2 as the dependent variable, and state well-being and meaning presence at Time 1 as the mediator, respectively. We found that meaning presence (but not state well-being) at Time 1 mediated cultural differences in state optimism at Time 2 (see Table 2 for the respective 95% percentile bootstrap confidence intervals of the indirect effects). For completeness, we also examined and found that (a) state well-being at Time 1 mediated the cultural differences in meaning presence at Time 2 and (b) meaning presence at Time 1 mediated the cultural differences in state well-being at Time 2 (see the last two rows in Table 2). We elaborate on the implications of these findings in Discussion.

## Discussion

The present research found that during the pandemic (March 2020), Chinese participants reported more positive affect and less negative affect, higher optimism, higher state well-being, and higher meaning presence, compared to Euro-Canadian participants. Chinese reported lower levels of general well-being than did Euro-Canadians, compatible with some prior studies with similar measures (e.g., Oishi, 2002). With a week’s interval between the two tests, the results were generally stable across time. Indeed, for each variable measured at both times, the test-retest correlation coefficients ranged between 0.67 and 0.76 (see Table 1). Furthermore, the relationships among different variables were stable, as similar patterns of results were observed at both times. As expected, we found that state optimism predicted, and mediated cultural differences in, subsequent state well-being and meaning presence. In addition, we found that state well-being and meaning presence also predicted subsequent state optimism, and that meaning presence (but not state well-being) mediated cultural differences in state optimism.

The present research shows that Chinese participants, while reporting lower life satisfaction in general, experienced more positive affect and less negative affect under the threat of the pandemic, compared to Euro-Canadian participants. These findings are consistent with prior work, which indicates that overall life satisfaction is influenced by cultural beliefs that are stable and chronic, whereas specific, online responses are subject to experiential and immediate contextual influences (e.g., the ups and downs that people are going through in their lives, Robinson and Clore, 2002). Our results also draw links with the literature on affective experiences (Uchida and Kitayama, 2009; Miyamoto and Ma, 2011) and the assumptions (Ji et al., 2001; Oishi, 2002) people have about life, satisfaction, and happiness, all of which can vary considerably across cultures.

More broadly, our mediation analyses with the longitudinal data (Table 2) revealed the temporal nature of key variables. That is, each of the three variables – optimism, well-being, meaning – at Time 1 mediated cultural differences in the other two variables at Time 2 (except that state well-being at Time 1 did not significantly mediate cultural differences in state optimism at Time 2). These results, compatible with past findings (Scheier and Carver, 1992; Wrosch and Scheier, 2003; Ho et al., 2010), have theoretical (e.g., incorporating time as an independent variable in theory-building) and practical (e.g., strategies for dealing with challenging events) implications for research on culture, coping, and health, as discussed below.

### Theoretical and Practical Implications

One feature that stands out in this research is its longitudinal design, which allows us to examine relationships among variables in temporal sequence. In growing fields such as cultural psychology where questions are as numerous as answers, longitudinal designs may provide insights that may otherwise be hidden. For example, longitudinal designs allow researchers to model time as an independent variable (Wright, 2007). Time, while assumed to play a role in many cultural processes (e.g., acculturation, lay theories of change, temporal focus, acquisition of norms), seldom gets integrated into research designs (see Barlett et al., 2014 for an exception). With a longitudinal view, researchers can systematically examine time as a causal, mediating, or moderating variable in cultural phenomena. This approach takes a step forward from standard studies in which cross-sectional data often capture a thin slice of fluid processes.

Furthermore, the present research reveals the mutual influence of state optimism, well-being, and meaning presence such that they predict one another over time. For example, state optimism at Time 1 accounted for cultural differences in state well-being or meaning presence at a Time 2, as state well-being or meaning presence at Time 1 accounted for cultural differences in state optimism at Time 2. The temporal impacts of these constructs on one another would have fallen out of our view if we had not collected data from the same participants at two different time points. The two waves of data collection were about one week apart during the pandemic. It is unclear to what extent the results would hold with a bigger temporal gap, which can be examined in future research.

Our mediational results suggest that optimism can help people go through challenges in life, leading to joy and meaning. Likewise, in difficult times, finding joy in small things and imbuing old routines with new purposes may result in a brighter outlook on life. Both scenarios resonate with the literature on health (Aspinwall and Taylor, 1992; Scheier and Carver, 1992; Slattery et al., 2017) and the non-linear theory of change (Ji et al., 2001, 2021). Furthermore, such reciprocal positive relationships can potentially lead to an upward spiral of empowerment, where state optimism, well-being, and sense of meaning perpetuate one another for good over time. Gradually, this cycle may become internalized, and people may be motivated to stay optimistic and imbue themselves with wellness and meaning for a positive, happy, and fulfilling life.

### Unpacking Cultural Differences: Conceptual Basis and Theoretical Links

The present research highlights differences in the responses to COVID-19 among Chinese and Euro-Canadians. Observed cultural patterns can be attributed to various cognitive and motivational processes, which were not examined directly due to the lack of resources during the pandemic. This is a limitation of the present research. Empirical demonstrations would have been more complete if we had the chance to measure naïve dialecticism, lay theories of change, cultural tightness-looseness, influence-adjustment motivations, and their respective roles in our results. These processes forge a theoretical ground for the present findings, as we discuss below.

Cross-cultural differences in optimism and well-being may be driven by naïve dialecticism (Peng and Nisbett, 1999), which assumes that the basis of life is full of contradictions, with good embedded in bad and bad embedded in good. Past research has shown that East Asians hold stronger beliefs in naïve dialecticism than North Americans (e.g., Nisbett, 2003; Spencer-Rodgers et al., 2010a). In particular, East Asians tend to embrace contradictions with high tolerance, whereas North Americans tend to view contradictions as something they should avoid in reasoning. Consistent with naïve dialecticism, prior work has shown that Chinese are more likely to infer the reality of things in a way that contradicts their public appearance, compared to Euro-Canadians (Ji et al., 2020; Lee et al., 2020). Related to naïve dialecticism, the non-linear theory of change (Ji, 2005) refers to the belief that the universe consists of opposing states, with everything in it constantly shifting from one state to another in a nonlinear fashion. For example, prosperity can change into poverty, and poverty can turn to prosperity. East Asians tend to hold a stronger belief than North Americans in the non-linear development of events. When predicting the future given past trends, Chinese participants tend to make predictions that deviate from the original propensity of the trend (e.g., a decreasing trend would go up), reflecting a non-linear theory of change. In contrast, Euro-American participants tend to make predictions that follow the propensity of the trend (e.g., a decreasing trend would keep going down), reflecting a linear theory of change (Ji et al., 2001, 2008).

Both naïve dialecticism and non-linear theory of change have implications when dealing with life adversities. These theories and beliefs, which people often take for granted thanks to cultural learning (e.g., Hirschfeld, 1996), exemplify how things in the world may not appear as they seem and how things may be opposite of what they appear to be. Within good there is evil, and beneath the surface of crisis there is opportunity for growth. Applying naïve dialecticism and non-linear theory of change to the context of COVID-19, one may predict that relative to Euro-Canadians, Chinese would be more inclined to react to the pandemic with positivity in terms of optimism, well-being, and meaning in life. This is indeed what our data show.

Another potential factor underlying the present findings is cultural tightness-looseness (Pelto, 1968; Gelfand et al., 2011), or the extent to which cultures vary in their tolerance for norm deviations and in their punishments for them. China, for instance, is considered as a tight culture, where social norms are closely followed and deviations from norms can easily result in sanctions by the group (Gelfand et al., 2011; Uz, 2015). In contrast, Canada and the U.S. are loose cultures, where most people do not expect sanctions by the group for not following social and cultural norms closely (Gelfand et al., 2011; Uz, 2015). Tightness and looseness across cultures, when applied to the current context, may provide another perspective as to why Chinese participants responded to the pandemic more positively than Euro-Canadian participants, as our results have shown. Rigid norms that stemmed from the pandemic—such as lock-down orders, masks, social distancing, and travel bans—are undeniably inconvenient to people. But these new norms, when viewed through the lens of tight cultures, are not as big of an issue because people in tight cultures, such as China, are strict followers of social norms on a regular basis. In contrast, in loose cultures, such as Canada, where norms are guides and deviations are common, people may have trouble adjusting to the new norms imposed abruptly onto their lives, especially the rigid norms in the COVID-19 pandemic that cannot be challenged. Cultural manifestations of tightness and looseness echo past work, which showed that East Asians are motivated to adjust themselves to the environment outside of them, whereas North Americans are motivated to influence the environment to fit them (Morling et al., 2002; Morling and Evered, 2006; Tsai et al., 2007).

Naïve dialecticism, lay theories of change, tightness-looseness, and adjustment-influence motivations are dimensions of culture that may contribute to how people react to the pandemic and the numerous safety rules that come with it. The respective and collective roles of these variables in pandemic-related reactions deserve a close look in future research, along with a broader and more gender-balanced sample.

### Beyond Negative Contexts?

The present research examined people’s responses in the context of a negative life experience. What would happen in a positive context? Applying cultural differences in reasoning styles, the opposite prediction might be made for positive life experience. For example, gratitude involves a positive life experience, as it represents “a felt sense of wonder, thankfulness and appreciation for life” (Emmons and Shelton, 2002, p. 460). Due to naïve dialecticism and non-linear theory of change, East Asians may generate negative responses while feeling gratitude. This prediction corresponds to cultural work on emotional complexity (Goetz et al., 2008; Miyamoto et al., 2010; An et al., 2017). It also contrasts with how gratitude is typically experienced by North Americans, which is overwhelmingly positive (Emmons and Shelton, 2002). The unconditional love of a friend can make people feel grateful, but also very guilty in some cultures, as some work has shown (e.g., Naito and Sakata, 2010). Likewise, Zhang et al. (2018) have shown that receiving verbal thanks may lead to stronger negative affective experiences among Chinese than among Euro-Canadians. In sum, the assumptions of dialecticism and lay theories of change may manifest in both negative and positive contexts. By examining this possibility, future research can enhance positive psychology with a cultural perspective.

### Limitations and Alternative Explanations

The present research would have been more complete in the presence of other measures that capture the way the pandemic was experienced by the participants, such as the level of threat perceived by our participants and possibly perceived knowledge of the virus. Due to limited time and resources, no such information was collected as the narrow window of opportunity was closing on us. Including these measures, and statistically controlling for them in our analyses, would have strengthened the present findings.

The present research was conducted during the pandemic. At the time of data collection, China (82,100 confirmed cases and 3,304 deaths on March 29, 2020)^5^ had more positive cases than Canada (6,258 cases and 63 deaths on March 29, 2020)^6^, but the trend was more concerning for Canada as cases there were on the rise while cases in China had reached a plateau. In addition, there may be differences between the two locations in terms of local policies imposed and the medical challenges faced at the time of this study. Thus, one may say that the two cultural groups were not exposed to the same levels of threat, which might have contributed to the results in some ways beyond our control. Our Chinese data were collected at a university in Wuhan, the city where the outbreak originated. Taking the viral impact at ground zero without prior warning, one may reasonably expect the looming terror of the pandemic to persist in Wuhan, even when the situation was somewhat under control by that point. The city was completely shut down for 2 months. In late March, city public transportations in Wuhan started to resume and some stores started to reopen. On April 8^*th*^, people in Wuhan were finally allowed to travel outside of the city, provided that they could show that they were virus-free with proper medical documents after 14 days of isolation. Still, residents were advised to stay home unless going out was absolutely necessary. Leaving and returning to their home compounds required examinations and documents. Students were learning online, while instructors were working off campus as well. Regardless of all the challenges, the situation was getting better overall. Meanwhile, in Kingston where the Canadian data were collected, the first 3 positive cases were identified on March 17, 2020, and were all travel related. The total positive cases were 43 on March 31, and 55 on April 14. Following the provincial declaration of a state of emergency on March 17, the city declared a city state of emergency on March 26. Students switched to online learning, and people were asked to work from home. Public transportation kept running. People could still travel, although the government encouraged people not to. Those who did travel were asked to self-quarantine for 14 days without any reinforcement by the government. Although the population density was low in Kingston and people were naturally more spread out against the threat of viral transmission, people had to change their behaviors and lifestyles, drastically and unprecedentedly, in anticipation of all kinds of uncertainties ahead. With different facts and realities in view, it is difficult to conclude which test location took a harder hit at the time of our study, though few would deny that both places were in bad shape. Still, we acknowledge the possibility that our cultural samples were experiencing different levels of threat from the pandemic, which could be a potential limitation of the present research.

## Conclusion

The present research examined the way people respond to the COVID-19 pandemic across cultures. Systematic cultural differences emerged in positive and negative affect, optimism, psychological well-being, and meaning presence. State optimism, well-being, and meaning presence not only reinforced each other, but also mediated cultural differences in one another over time. These findings may shed new light on theoretical development and generate practical implications for psychological health and well-being in real life.

## Data Availability Statement

The raw data supporting the conclusions of this article will be made available by the authors, without undue reservation.

## Ethics Statement

The studies involving human participants were reviewed and approved by the General Research Ethics Board, Queen’s University. The patients/participants provided their written informed consent to participate in this study.

## Author Contributions

L-JJ and SY conceived the research idea, designed the study, and analyzed the data. SY, YL, and YD collected the data. AL did the literature review and wrote the manuscript with L-JJ. All authors undertook final clarification and agreed on the version of the manuscript for submission.

## Conflict of Interest

The authors declare that the research was conducted in the absence of any commercial or financial relationships that could be construed as a potential conflict of interest.

## Footnotes

1 The empirical picture of cultural differences in trait optimism is less coherent. On the one hand, research using standard measures of trait optimism (e.g., Extended Life Orientation Test; Chang et al., 1997) have shown that Euro-Americans are more optimistic compared to East Asians (Chang, 1996). Similar conclusions (Heine and Lehman, 1995; Lee and Seligman, 1997) appeared in other work with different measurements (e.g., attributional styles, scenarios). On the other hand, no cultural difference in optimism was observed in some studies (Ji et al., 2004; see Fischer and Chalmers, 2008 for a meta-analysis). Possible explanations have been proposed, including the cultural meanings of optimism (Lai and Yue, 2000), multi-faceted nature of the construct (Ji et al., 2004), presence of confounds (Chang, 1996), and the impacts of contextual information (Ji et al., 2004, 2021). In light of these discussions, we were unable to make specific predictions about cultural differences in trait optimism but decided to include it for the sake of completion.

2 During the time of the study, both locations were under quarantine.

3 Participants were asked to generate a unique ID, which was used to match their data across time. Unfortunately, some participants at Time 2 did not follow instruction or provided a different ID and, thus, were not matched over time.

4 We also measured resilience with the 4-item Brief Resilience Coping Scale (Sinclair and Wallston, 2004). Unfortunately, the scale had low internal consistency and did not establish measurement invariance across cultures, and thus was excluded from the report in this paper. See details in the Supplementary Material.

5 https://www.statista.com/statistics/1092918/china-wuhan-coronavirus-2019ncov-confirmed-and-deceased-number/

6 https://www.canada.ca/en/public-health/services/diseases/2019-novel-coronavirus-infection.html?topic=tilelink

## References

[B1] Aglozo E. Y. Akotia C. S. Osei-Tutu A. Annor F. (2019). Spirituality and subjective well-being among Ghanaian older adults: optimism and meaning in life as mediators. *Aging Ment. Health* 25 306–315. 10.1080/13607863.2019.1697203 31814428

[B2] An S. Ji L. J. Marks M. Zhang Z. (2017). Two sides of emotion: exploring positivity and negativity in six basic emotions across cultures. *Front. Psychol.* 8:610. 10.3389/fpsyg.2017.00610 28473791 PMC5397534

[B3] Aspinwall L. G. Taylor S. E. (1992). Modeling cognitive adaptation: a longitudinal investigation of the impact of individual differences and coping on college adjustment and performance. *J. Pers. Soc. Psychol.* 63 989–1003. 10.1037/0022-3514.63.6.989 1460565

[B4] Bagozzi R. P. Wong N. Yi Y. (1999). The role of culture and gender in the relationship between positive and negative affect. *Cogn. Emot.* 13 641–672. 10.1080/026999399379023

[B5] Baltes P. B. Staudinger U. M. (2000). Wisdom: a metaheuristic (pragmatic) to orchestrate mind and virtue toward excellence. *Am. Psychol.* 55 122–136. 10.1037//0003-066X.55.1.122 11392856

[B6] Barlett C. P. Gentile D. A. Anderson C. A. Suzuki K. Sakamoto A. Yamaoka A. (2014). Cross-cultural differences in cyberbullying behavior: a short-term longitudinal study. *J. Cross Cult. Psychol.* 45 300–313. 10.1177/0022022113504622

[B7] Battista J. Almond R. (1973). The development of meaning in life. *Psychiatry* 36 409–427. 4756415 10.1080/00332747.1973.11023774

[B8] Bech P. Gudex C. Johansen S. (1996). The WHO (ten) well-being index: validation in diabetes. *Psychother. Psychosom.* 65 183–190. 10.1159/000289073 8843498

[B9] Birren J. E. Svensson C. M. (2005). “Wisdom in history,” in *A Handbook of Wisdom: Psychological Perspectives*, eds Sternberg R. J. Jordan J. (New York, NY: Cambridge University Press), 3–28.

[B10] Bruehlman-Senecal E. Ayduk Ö John O. P. (2016). Taking the long view: implications of individual differences in temporal distancing for affect, stress reactivity, and well-being. *J. Pers. Soc. Psychol.* 111 610–635. 10.1037/pspp0000103 27399251

[B11] Carvajal S. C. Clair S. D. Nash S. G. Evans R. I. (1998). Relating optimism, hope, and self-esteem to social influences in deterring substance use in adolescents. *J. Soc. Clin. Psychol.* 17 443–465. 10.1521/jscp.1998.17.4.443

[B12] Carver C. S. Peterson L. M. Follansbee D. J. Scheier M. F. (1983). Effects of self-directed attention on performance and persistence among persons high and low in test anxiety. *Cogn. Ther. Res.* 7 333–353. 10.1007/BF01177556

[B13] Carver C. S. Scheier M. F. Segerstrom S. C. (2010). Optimism. *Clin. Psychol. Rev.* 30 879–889. 10.1016/j.cpr.2010.01.006 20170998 PMC4161121

[B14] Chang E. C. (1996). Cultural differences in optimism, pessimism, and coping: predictors of subsequent adjustment in Asian American and Caucasian American college students. *J. Couns. Psychol.* 43 113–123.

[B15] Chang E. C. Maydeu-Olivares A. D’Zurilla T. J. (1997). Optimism and pessimism as partially independent constructs: relationship to positive and negative affectivity and psychological well-being. *Pers. Individ. Differ.* 23 433–440. 10.1016/S0191-8869(97)80009-8

[B16] Cheng C. (2009). Dialectical thinking and coping flexibility: a multimethod approach. *J. Pers.* 77 471–493. 19220723 10.1111/j.1467-6494.2008.00555.x

[B17] Chow P. I. Berenbaum H. (2012). Perceived utility of emotion: the structure and construct validity of the perceived affect utility scale in a cross-ethnic sample. *Cultur. Divers. Ethnic Minor. Psychol.* 18 55–63. 22250898 10.1037/a0026711

[B18] Cohen D. Hoshino-Browne E. Leung A. K.-y (2007). “Culture and the structure of personal experience: insider and outsider phenomenologies of the self and social world,” in *Advances in Experimental Social Psychology*, Vol. 39 ed. Zanna M. P. (San Diego, CA: Elsevier), 1–67. 10.1016/S0065-2601(06)39001-6

[B19] Cooke P. J. Melchert T. P. Connor K. (2016). Measuring well-being: a review of instruments. *Couns. Psychol.* 44 730–757. 10.1177/0011000016633507

[B20] Daukantaite D. Bergman L. R. (2005). Childhood roots of women’s subjective well-being: the role of optimism. *Eur. Psychol.* 10 287–297. 10.1027/1016-9040.10.4.287

[B21] Dember W. N. Martin S. H. Hummer M. K. Howe S. R. Melton R. S. (1989). The measurement of optimism and pessimism. *Curr. Psychol.* 8 102–119. 10.1007/BF02686675

[B22] Diener E. (2009). Subjective well-being. *Psychol. Bull.* 95 542–575. 6399758

[B23] Diener E. D. Emmons R. A. Larsen R. J. Griffin S. (1985). The satisfaction with life scale. *J. Pers. Assess.* 49 71–75. 16367493 10.1207/s15327752jpa4901_13

[B24] Duffy R. D. Bott E. M. Allan B. A. Torrey C. L. (2013). Examining a model of life satisfaction among unemployed adults. *J. Couns. Psychol.* 60 53–63. 10.1037/a0030771 23163613

[B25] Dyer J. G. McGuinness T. M. (1996). Resilience: analysis of the concept. *Arch. Psychiatr. Nurs.* 10 276–282. 10.1016/S0883-9417(96)80036-7 8897710

[B26] Emmons R. A. Shelton C. M. (2002). “Gratitude and the science of positive psychology,” in *Handbook of Positive Psychology*, eds Snyder C. R.,and Lopez S. J. (Oxford: Oxford University Press), 459–471.

[B27] Fawcett J. Scheftner W. Clark D. Hedeker D. Gibbons R. Coryell W. (1987). Clinical predictors of suicide in patients with major affective disorders: a controlled prospective study. *Am. J. Psychiatry* 144 35–40. 10.1176/ajp.144.1.35 3799837

[B28] Fischer R. Chalmers A. (2008). Is optimism universal? A meta-analytical investigation of optimism levels across 22 nations. *Pers. Individ. Differ.* 45 378–382. 10.1016/j.paid.2008.05.008

[B29] Fournier M. De Ridder D. Bensing J. (2002). Optimism and adaptation to chronic disease: the role of optimism in relation to self-care options of type 1 diabetes mellitus, rheumatoid arthritis and multiple sclerosis. *Br. J. Health Psychol.* 7(Pt 4) 409–432. 10.1348/135910702320645390 12614494

[B30] Frazier P. A. Gavian M. Hirai R. Park C. Tennen H. Tomich P. (2011). Prospective predictors of posttraumatic stress disorder symptoms: direct and mediated relations. *Psychol. Trauma Theory Res. Pract. Policy* 3 27–36. 10.1037/a0019894

[B31] Friedman L. C. Nelson D. V. Baer P. E. Lane M. Smith F. E. Dworkin R. J. (1992). The relationship of dispositional optimism, daily life stress, and domestic environment to coping methods used by cancer patients. *J. Behav. Med.* 15 127–141. 10.1007/BF00848321 1583677

[B32] Gelfand M. J. Raver J. L. Nishii L. Leslie L. M. Lun J. Lim B. C. (2011). Differences between tight and loose cultures: a 33-nation study. *Science* 332 1100–1104. 21617077 10.1126/science.1197754

[B33] Goetz J. L. Spencer-Rodgers J. Peng K. (2008). “Dialectical emotions,” in *Handbook of Motivation and Cognition Across Cultures*, eds Sorrentino R. Yamguchi S. (London: Elsevier), 517–539. 10.1016/B978-0-12-373694-9.00022-2

[B34] Grossmann I. Huynh A. C. Ellsworth P. C. (2016). Emotional complexity: clarifying definitions and cultural correlates. *J. Pers. Soc. Psychol.* 111 895–916. 26692354 10.1037/pspp0000084

[B35] Grossmann I. Karasawa M. Kan C. Kitayama S. (2014). A cultural perspective on emotional experiences across the life span. *Emotion* 14 679–692. 24749641 10.1037/a0036041

[B36] Grossmann I. Na J. Varnum M. E. W. Kitayama S. Nisbett R. E. (2013). A route to well-being: intelligence versus wise reasoning. *J. Exp. Psychol. Gen.* 142 944–953. 10.1037/a0029560 22866683 PMC3594053

[B37] Halama P. Dedova M. (2007). Meaning in life and hope as predictors of positive mental health: do they explain residual variance not predicted by personality traits? *Studia Psychol.* 49 191–200.

[B38] Heine S. J. Lehman D. R. (1995). Cultural variation in unrealistic optimism: does the West feel more vulnerable than the East? *J. Pers. Soc. Psychol.* 68 595–607. 10.1037/0022-3514.68.4.595

[B39] Hirschfeld L. A. (1996). *Race in the Making: Cognition, Culture, and the Child’s Construction of Human Kinds.* Cambridge, MA: MIT Press.

[B40] Ho M. Y. Cheung F. M. Cheung S. F. (2010). The role of meaning in life and optimism in promoting well-being. *Pers. Individ. Differ.* 48 658–663. 10.1016/j.paid.2010.01.008

[B41] Hou Y. Zhu Y. Peng K. (2003). Thinking style and disease cognitions among chinese people. *J. Psychol. Chinese Soc.* 4 161–180.

[B42] Ji L. J. (2005). “Culture and lay theories of change,” in *Cultural and Social Behavior: The Ontario Symposium*, Vol. 10 eds Sorrentino R. M. Cohen D. Olson J. M. Zanna M. P. (Mahwah, NJ: Lawrence Erlbaum Associates Publishers), 117–135.

[B43] Ji L. J. (2008). The leopard cannot change his spots, or can he: culture and the development of lay theories of change. *Pers. Soc. Psychol. Bull.* 34 613–622. 18322267 10.1177/0146167207313935

[B44] Ji L. J. Guo T. Zhang Z. Messervey D. (2009). Looking into the past: cultural differences in perception and representation of past information. *J. Pers. Soc. Psychol.* 96 761–769. 19309200 10.1037/a0014498

[B45] Ji L. J. Hong E. Guo T. Zhang Z. Su Y. Li Y. (2019). Culture, psychological proximity to the past and future, and self-continuity. *Eur. J. Soc. Psychol.* 49 735–747.

[B46] Ji L. J. Khei M. Yap S. Wang X. Zhang Z. Hou Y. (2020). Cultural differences in the construal of suffering and the Covid-19 pandemic. *Soc. Psychol. Pers. Sci.* 194855062095880. 10.1177/1948550620958807

[B47] Ji L. J. Lee A. Guo T. (2010). “The thinking styles of Chinese people,” in *The Oxford Handbook of Chinese Psychology*, ed. Bond M. H. (New York, NY: Oxford University Press), 155–167.

[B48] Ji L. J. Nisbett R. E. Su Y. (2001). Culture, change, and prediction. *Psychol. Sci.* 12 450–456. 10.1111/1467-9280.00384 11760130

[B49] Ji L. J. Vaughen-Johnson, Zhang Z. Jacobson J. Zhang N. Huang X. (2021). Context and cultural differences in optimism. *J. Cross Cult. Psychol.* 52 449–467. 10.1177/00220221211020442

[B50] Ji L. J. Peng K. Nisbett R. (2000). Culture, control, and perception of relationships in the environment. *J. Pers. Soc. Psychol.* 78 943–955. 10.1037/0022-3514.78.5.943 10821200

[B51] Ji L. J. Zhang Z. Guo T. (2008). To buy or to sell: cultural differences in stock market decisions based on price trends. *J. Behav. Decis. Making* 21 399–413. 10.1002/bdm.595

[B52] Ji L. J. Zhang Z. Usborne E. Guan Y. (2004). Optimism across cultures: in response to the severe acute respiratory syndrome outbreak: SARS: optimism across cultures. *Asian J. Soc. Psychol.* 7 25–34. 10.1111/j.1467-839X.2004.00132.x

[B53] Kealy D. Ben-David S. Cox D. W. (2020). Early parental support and meaning in life among young adults: the mediating roles of optimism and identity. *Curr. Psychol.* 10.1007/s12144-020-00907-w Advance online publication

[B54] Kitayama S. Markus H. R. Kurokawa M. (2000). Culture, emotion, and well-being: good feelings in Japan and the United States. *Cogn. Emot.* 14 93–124.

[B55] Klinger E. (1977). *Meaning and Void.* Minneapolis, Min: University of Minnesota Press.

[B56] Krause N. (2003). Religious meaning and subjective well-being in late life. *J. Gerontol. Ser. B* 58 160–170. 10.1093/geronb/58.3.S160 12730317

[B57] Lai J. C. L. Yue X. D. (2000). Measuring optimism in Hong Kong and Mainland Chinese with the revised Life Orientation Test. *Pers. Individ. Differ.* 28 781–796. 10.1016/S0191-8869(99)00138-5

[B58] Lee A. Ji L.-J. Li Y. Zhang Z. (2020). Fear Goliath or David? Inferring competence from demeanor across cultures. *Pers. Soc. Psychol. Bull.* 46 1074–1089. 10.1177/0146167219893999 31889473

[B59] Lee Y.-T. Seligman M. E. P. (1997). Are Americans more optimistic than the Chinese? *Pers. Soc. Psychol. Bull.* 23 32–40. 10.1177/0146167297231004

[B60] Masten A. S. Best K. M. Garmezy N. (1990). Resilience and development: contributions from the study of children who overcome adversity. *Dev. Psychopathol.* 2 425–444. 10.1017/S0954579400005812

[B61] Millstein R. A. Chung W.-J. Hoeppner B. B. Boehm J. K. Legler S. R. Mastromauro C. A. (2019). Development of the state optimism measure. *Gen. Hosp. Psychiatry* 58 83–93. 10.1016/j.genhosppsych.2019.04.002 31026732 PMC6501845

[B62] Miyamoto Y. Ma X. (2011). Dampening or savoring positive emotions: a dialectical cultural script guides emotion regulation. *Emotion* 11 1346–1357. 21910543 10.1037/a0025135

[B63] Miyamoto Y. Uchida Y. Ellsworth P. C. (2010). Culture and mixed emotions: co-occurrence of positive and negative emotions in Japan and the United States. *Emotion* 10 404–415. 20515228 10.1037/a0018430

[B64] Morling B. Evered S. (2006). Secondary control reviewed and defined. *Psychol. Bull.* 132 269–296. 16536644 10.1037/0033-2909.132.2.269

[B65] Morling B. Kitayama S. Miyamoto Y. (2002). Cultural practices emphasize influence in the United States and adjustment in Japan. *Pers. Soc. Psychol. Bull.* 28 311–323.

[B66] Morris M. W. Peng K. (1994). Culture and cause: American and Chinese attributions for social and physical events. *J. Pers. Soc. Psychol.* 67 949–971. 10.1037/0022-3514.67.6.949

[B67] Naito T. Sakata Y. (2010). Gratitude, indebtedness, and regret on receiving a friend’s favor in Japan. *Psychologia* 53 179–194. 10.2117/psysoc.2010.179

[B68] Napa Scollon C. Diener E. Oishi S. Biswas-Diener R. (2005). An experience sampling and cross-cultural investigation of the relation between pleasant and unpleasant affect. *Cogn. Emot.* 19 27–52.

[B69] Nisbett R. E. (2003). *The Geography of Thought: How Asians and Westerners Think Differently– and Why.* New York, NY: Free Press.

[B70] O’Leary V. E. Ickovics J. R. (1995). Resilience and thriving in response to challenge: an opportunity for a paradigm shift in women’s health. *Womens Health* 1 121–142. 9373376

[B71] Oishi S. (2002). The experiencing and remembering of well-being: a cross-cultural analysis. *Pers. Soc. Psychol. Bull.* 28 1398–1406. 10.1177/014616702236871

[B72] Pelto P. (1968). The difference between “tight” and “loose” societies. *Transaction* 5 37–40.

[B73] Peng K. Nisbett R. E. (1999). Culture, dialectics, and reasoning about contradiction. *Am. Psychol.* 54 741–754. 10.1037/0003-066X.54.9.741

[B74] Prasso S. (2020). *China’s Divorce Spike is a Warning to Rest of Locked-Down World.* Bloomberg Businessweek. Available online at: https://www.bloomberg.com/news/articles/2020-03-31/divorces-spike-in-china-after-coronavirus-quarantines (accessed March 31, 2020).

[B75] R Core Team (2019). *R: A Language and Environment for Statistical Computing.* Vienna: R Foundation for Statistical Computing.

[B76] Robbins A. S. Spence J. T. Clark H. (1991). Psychological determinants of health and performance: the tangled web of desirable and undesirable characteristics. *J. Pers. Soc. Psychol.* 61 755–765. 10.1037/0022-3514.61.5.755 1753330

[B77] Robinson M. D. Clore G. L. (2002). Belief and feeling: evidence for an accessibility model of emotional self-report. *Psychol. Bull.* 128 934–960. 12405138 10.1037/0033-2909.128.6.934

[B78] Rosseel Y. (2012). lavaan: an R package for structural equation modeling. *J. Stat. Softw.* 48 1–36. 10.18637/jss.v048.i02

[B79] Ryan R. M. Deci E. L. (2001). On happiness and human potentials: a review of research on hedonic and eudaimonic well-being. *Annu. Rev. Psychol.* 52 141–166. 10.1146/annurev.psych.52.1.141 11148302

[B80] Ryff C. D. Keyes C. L. M. (1995). The structure of psychological well-being revisited. *J. Pers. Soc. Psychol.* 69 719–727. 10.1037/0022-3514.69.4.719 7473027

[B81] Ryff C. D. Singer B. (1998). The contours of positive human health. *Psychol. Inq.* 9 1–28.

[B82] Scheier M. F. Carver C. S. (1985). Optimism, coping, and health: assessment and implications of generalized outcome expectancies. *Health Psychol.* 4 219–247. 10.1037/0278-6133.4.3.219 4029106

[B83] Scheier M. F. Carver C. S. (1992). Effects of optimism on psychological and physical well-being: theoretical overview and empirical update. *Cogn. Ther. Res.* 16 201–228. 10.1007/BF01173489

[B84] Scheier M. F. Carver C. S. (2018). Dispositional optimism and physical health: a long look back, a quick look forward. *Am. Psychol.* 73 1082–1094. 10.1037/amp0000384 30525784 PMC6309621

[B85] Scheier M. F. Carver C. S. Bridges M. W. (1994). Distinguishing optimism from neuroticism (and trait anxiety, self-mastery, and self-esteem): a reevaluation of the Life Orientation Test. *J. Pers. Soc. Psychol.* 67 1063–1078. 10.1037//0022-3514.67.6.1063 7815302

[B86] Sinclair V. G. Wallston K. A. (2004). The development and psychometric evaluation of the brief resilient coping scale. *Assessment* 11 94–101. 10.1177/1073191103258144 14994958

[B87] Slattery É McMahon J. Gallagher S. (2017). Optimism and benefit finding in parents of children with developmental disabilities: the role of positive reappraisal and social support. *Res. Dev. Disabil.* 65 12–22. 10.1016/j.ridd.2017.04.006 28432893

[B88] Spencer-Rodgers J. Peng K. Wang L. (2010a). Dialecticism and the co-occurrence of positive and negative emotions across cultures. *J. Cross Cult. Psychol.* 41 109–115.

[B89] Spencer-Rodgers J. Williams M. J. Peng K. (2010b). Cultural differences in expectations of change and tolerance for contradiction: a decade of empirical research. *Pers. Soc. Psychol. Rev.* 14 296–312. 10.1177/1088868310362982 20435801

[B90] Steger M. F. Frazier P. Oishi S. Kaler M. (2006). The meaning in life questionnaire: assessing the presence of and search for meaning in life. *J. Couns. Psychol.* 53 80–93.

[B91] Sun F. (2020). *Covid-19 Toll on Marriage: Divorce Inquiries on the Rise as Stay-Home Measures Push Hong Kong Couples Off the Edge.* South China Morning Post. Available online at: https://www.scmp.com/news/hong-kong/health-environment/article/3083681/covid-19-toll-marriage-divorce-inquiries-rise (accessed May 10, 2020).

[B92] Tsai J. L. Miao F. F. Seppala E. Fung H. H. Yeung D. Y. (2007). Influence and adjustment goals: sources of cultural differences in ideal affect. *J. Pers. Soc. Psychol.* 92 1102–1117. 17547491 10.1037/0022-3514.92.6.1102

[B93] Uchida Y. Kitayama S. (2009). Happiness and unhappiness in East and West: themes and variations. *Emotion* 9 441–456. 19653765 10.1037/a0015634

[B94] Uz I. (2015). The index of cultural tightness and looseness among 68 countries. *J. Cross Cult. Psychol.* 46 319–335.

[B95] Wray T. B. Dvora R. D. Hsia J. F. Arens A. M. Schweinle W. E. (2013). Optimism and pessimism as predictors of alcohol use trajectories in adolescence. *J. Child Adolesc. Subst. Abuse* 22 58–68. 10.1080/1067828X.2012.729915

[B96] Wright T. A. (2007). “A look at two methodological challenges for scholars interested in positive organizational behavior,” in *Positive Organizational Behavior: Accentuating the Positive at Work*, eds Nelson D. Cooper C. L. (Thousand Oaks, CA: SAGE Publications Ltd), 177–190. 10.4135/9781446212752.n13

[B97] Wrosch C. Scheier M. F. (2003). Personality and quality of life: the importance of optimism and goal adjustment. *Qual. Life Res.* 12(Suppl. 1) 59–72. 12803312 10.1023/a:1023529606137

[B98] Wu S. Keysar B. (2007). The effect of culture on perspective taking. *Psychol. Sci.* 18 600–606. 10.1111/j.1467-9280.2007.01946.x 17614868

[B99] Yamamoto J. I. (1998). *Buddhism, Taoism, and other far Eastern Religions (No. 6).* Grand Rapids, MI: Zondervan.

[B100] Yu E. A. Chang E. C. (2019). Meaning in life as a predictor of optimism: how parents mattering matters to Asian and European Americans. *Pers. Individ. Differ.* 138 366–369. 10.1016/j.paid.2018.10.031

[B101] Yzerbyt V. Muller D. Batailler C. Judd C. M. (2018). New recommendations for testing indirect effects in mediational models: the need to report and test component paths. *J. Pers. Soc. Psychol.* 115 929–943. 10.1037/pspa0000132 30550319

[B102] Zhang N. Ji L. J. Bai B. Li Y. (2018). Culturally divergent consequences of receiving thanks in close relationships. *Emotion* 18 46–57. 10.1037/emo0000385 29172616

