# Supplementary material for: Cultural Differences in People’s Psychological Response to COVID-19
Source: Front Psychol. 2021 Jul 12;12:636062. doi: 10.3389/fpsyg.2021.636062 (PMC8312224; doi:10.3389/fpsyg.2021.636062)
Supplement: Supplementary file 1 [file Data_Sheet_1.pdf]

Supplementary Material

1. **Measurement invariance tests based on data from Time 1.**

In general, we have made decisions on model fit holistically based on the following criteria in evaluating the model fit indices. The specific cutoff values were used following previous research (Chen, 2007; Hu & Bentler, 1999; Putnick & Bornstein, 2016). RMSEA estimates can be artificially high for models with low degrees of freedom, so if other indices were acceptable but RMSEA was not in models with small dfs, we decided to accept the model. Furthermore, among all other indices (besides RMSEA), if most of them were acceptable while one or two were marginal (e.g., .033 instead of .03), we decided to accept the model.

Table S1. Model fit criteria.

CFI	RMSEA (90%CI)	SRMR	ΔCFI	ΔRMSEA	ΔSRMR
.90	.08	.08	.01	.015	.030 (for metrics invariance)<br>.015 (for scalar invariance)

Chen, F. F. (2007). Sensitivity of goodness of fit indexes to lack of measurement invariance. *Structural Equation Modeling*, *14*, 464–504. doi:10.1080/10705510701301834.

Putnick, D. L., & Bornstein, M. H. (2016). Measurement invariance conventions and reporting: The state of the art and future directions for psychological research. *Developmental review*, *41*, 71-90,

Hu, L. & Bentler, P. (1999). Cutoff criteria for fit indices in covariance structure analysis: conventional criteria versus new alternatives. *Structural Equation Modeling, 6*, 1-55.

When the full scale did not establish measure invariance, we then examined the factor loadings and item intercepts on an item-by-item basis to determine which items were the main contributors toward measurement noninvariance. We followed this procedure to trim down the items for each measure.

A. Measurement invariance tests: adapted WHO-10

Table S2: Measurement invariance tests for Adapted WHO-10 with all 10 items.

Model	χ^2 (df)	CFI	RMSEA	SRMR	Δχ2 (Δdf)	ΔCFI	ΔRMSEA	ΔSRMR	Decision
Config	208.80(70)	.944	.085	.041					accept
Metrics (loading)	244.86(79)	.933	.087	.075	35.06(9); *p*<.0001	.011	.002	.033	accept
Scalar (intercept)	507.95(88)	.830	.131	.114	263.09(9); *p*<.0001	.103	.044	.039	reject

Partial measurement invariance (configural, and metric, and scalar) was established with 5 of the 10 items (1, 3, 4, 6, 10). Note: RMSEA is higher than ideal, but the other indices suggest a decent fit.

Table S3: Measurement invariance tests for Adapted WHO-10 with 5 items.

Model	χ^2 (df)	CFI	RMSEA	SRMR	Δχ2 (Δdf)	ΔCFI	ΔRMSEA	ΔSRMR	Decision
Config	40.63(10)	.968	.105	.033					accept
Metrics (loading)	56.15(14)	.956	.104	.065	15.52 (4); *p*=.0037	.012	.001	.033	accept
Scalar (intercept)	61.50(18)	.954	.093	.068	5.35 (4); *p*=.254	.001	.011	.003	accept

B. SWLS

Table S4: Measurement invariance tests for SWLS with all 5 items.

Model	χ^2 (df)	CFI	RMSEA	SRMR	Δχ2 (Δdf)	ΔCFI	ΔRMSEA	ΔSRMR	Decision
Config	39.40(10)	.979	.103	.027					accept
Metrics (loading)	53.97(14)	.972	.101	.058	14.57(4); *p*=.006	.007	.001	.031	accept
Scalar (intercept)	223.89(18)	.854	.202	.117	169.92(4); *p*<.0001	.118	.101	.059	reject

Partial measurement invariance (configural, and metric) was established with 4 of the 5 items (1-3, 5), as well as with all 5 items. Dropping items did not improve measure invariance.

Table S5: Measurement invariance tests for SWLS with 4 items.

Model	χ^2 (df)	CFI	RMSEA	SRMR	Δχ2 (Δdf)	ΔCFI	ΔRMSEA	ΔSRMR	Decision
Config	17.22(4)	.99	.109	.019					accept
Metrics (loading)	25.30(7)	.98	.097	.047	8.07(3); *p*=.044	.005	.012	.029	accept
Scalar (intercept)	83.28(10)	.93	.162	.091	57.98(3); *p*<.001	.054	.065	.043	reject

C. State Optimism (SOM-7)

Table S6: Measurement invariance tests for SOM-7 with all 7 items.

Model	χ^2 (df)	CFI	RMSEA	SRMR	Δχ2(Δdf)	ΔCFI	ΔRMSEA	ΔSRMR	Decision
Config	69.42(28)	.980	.073	.027					accept
Metrics (loading)	82.04(34)	.976	.071	.048	12.62(6); p=.049	.003	.002	.020	accept

Scalar (intercept)	161.10(40)	.940	.104	.063	79.05 (6); p<.0001	.036	.033	.016	reject

Partial measurement invariance (configural, metric, and scalar) was established with 6 of the 7 items (1-6).

Table S7: Measurement invariance tests for SOM-7 with 6 items.

Model	χ^2 (df)	CFI	RMSEA	SRMR	Δχ2(Δdf)	ΔCFI	ΔRMSEA	ΔSRMR	Decision
Config	42.31(18)	.984	.070	.026					accept
Metrics (loading)	50.53(23)	.982	.066	.043	8.22(5); *p*=.145	.002	.004	.017	accept
Scalar (intercept)	58.20(28)	.980	.062	.046	7.67(5); *p*=.175	.002	.003	.004	accept

D. Resilience (BRC)

Resilience was measured with the 4-item Brief Resilience Coping Scale (BRC; Sinclaire & Wallston, 2004). Participants indicated (1 = *does not describe me at all*, 5 = *describes me very well*) how well each of the four statements described their actions "*these days*" (e.g., "*I believe I can grow in positive ways by dealing with difficult situations*").

Table S8: Measurement invariance tests for BRC with all 4 items.

Model	χ^2 (df)	CFI	RMSEA	SRMR	Δχ2 (Δdf)	ΔCFI	ΔRMSEA	ΔSRMR	Decision
Config	18.42(4)	.954	.114	.034					accept
Metrics (loading)	26.28(7)	.938	.100	.052	7.86(3); *p*=.049	.016	.014	.017	accept?
Scalar (intercept)	119.45(10)	.650	.199	.116	93.17(3); *p*<.001	.289	.099	.065	reject

Dropping any one of the 4 items did not improve the model fit. Among all 3-item models, the following (with items 2-4) provided the best indices, but the overall model fit was not better than that with all 4 items, and internal consistency of the 3-item models dropped to the .4-.5 range.

Table S9: Measurement invariance tests for BRC with 3 items.

Model	χ^2 (df)	CFI	RMSEA	SRMR	Δχ2 (Δdf)	ΔCFI	ΔRMSEA	ΔSRMR	Decision
Config	0 (0)	1.00	.000	.000					accept
Metrics (loading)	3.13(2)	.99	.045	.024	3.13(2); *p*=.209	.007	.045	.024	reject

Scalar (intercept)	76.02(4)	.56	.260	.126	76.02(2); *p*<.001	.430	.215	.101	reject

E. Meaning in life

Table S10: Measurement invariance tests for MIL with all 10 items.

Model	χ^2 (df)	CFI	RMSEA	SRMR	Δχ2 (Δdf)	ΔCFI	ΔRMSEA	ΔSRMR	Decision
Config	203.88(68)	.950	.085	.059					accept
Metrics (loading)	217.32(76)	.948	.082	.065	13.44 (8); *p*=.097	.002	.003	.005	accept
Scalar (intercept)	314.82 (84)	.916	.100	.076	97.50 (8); *p*<.0001	.033	.018	.012	reject

Partial measurement invariance (configural, metric, and scalar) was established with 8 of the 10 items (1,3-8, 10).

Table S11: Measurement invariance tests for MIL with 8 items.

Model	χ^2 (df)	CFI	RMSEA	SRMR	Δχ2 (Δdf)	ΔCFI	ΔRMSEA	ΔSRMR	Decision
Config	97.32(38)	.972	.075	.051					accept
Metrics (loading)	105.52(44)	.971	.071	.058	8.20 (6), *p*=.224	.001	.004	.007	accept
Scalar (intercept)	136.53(50)	.959	.079	.064	31.05(6); *p*<.001	.012	.008	.005	accept

F. LOT-R

Table S12: Measurement invariance tests for LOT-R with all 6 items.

Model	χ^2 (df)	CFI	RMSEA	SRMR	Δχ2 (Δdf)	ΔCFI	ΔRMSEA	ΔSRMR	Decision
Config	108.14(18)	.893	.134	.062					reject
Metrics (loading)	148.66(23)	.850	.140	.090	40.52 (5); *p*<.0001	.042	.006	.027	reject
Scalar (intercept)	343.47(28)	.625	.201	.143	194.81(5); *p*<.0001	.226	.061	.053	reject

Arguably partial measurement invariance (configural) was established with 4 of the 6 items (3-6), but it was not better than the full scale.

Table S13: Measurement invariance tests for LOT-R with 4 items.

Model	χ^2 (df)	CFI	RMSEA	SRMR	Δχ2 (Δdf)	ΔCFI	ΔRMSEA	ΔSRMR	Decision
Config	15.70 (4)	.977	.102	.034					accept?
Metrics (loading)	40.11 (7)	.936	.130	.074	24.40 (3); *p*<.0001	.042	.028	.041	reject
Scalar (intercept)	138.47(10)	.750	.214	.123	98.36 (3); *p*<.0001	.186	.084	.048	reject

2. **Measurement invariance tests based on data from Time 2**

G. Measurement invariance tests: adapted WHO-10

Table S14: Measurement invariance tests for Adapted WHO-10 with all 10 items.

Model	χ^2 (df)	CFI	RMSEA	SRMR	Δχ2 (Δdf)	ΔCFI	ΔRMSEA	ΔSRMR	Decision
Config	179.16(70)	.951	.081	.040					accept
Metrics (loading)	213.12(79)	.939	.084	.076	33.96(9); *p*<.0001	.011	.004	.036	accept?
Scalar (intercept)	345.04(88)	.884	.111	.099	131.92(9); *p*<.0001	.056	.026	.023	reject

Partial measurement invariance (configural, and metric, and scalar) was established with 5 of the 10 items (1, 3, 4, 6, 10).

Table S15: Measurement invariance tests for Adapted WHO-10 with 5 items.

Model	χ^2 (df)	CFI	RMSEA	SRMR	Δχ2 (Δdf)	ΔCFI	ΔRMSEA	ΔSRMR	Decision
Config	30.31(10)	.974	.092	.031					accept
Metrics (loading)	34.76(14)	.973	.079	.044	4.45 (4); *p*=.349	.001	.013	.013	accept
Scalar (intercept)	48.47(18)	.960	.084	.053	13.72 (4); *p*=.008	.013	.005	.010	accept

H. SWLS

Table S16: Measurement invariance tests for SWLS with all 5 items.

Model	χ^2 (df)	CFI	RMSEA	SRMR	Δχ2 (Δdf)	ΔCFI	ΔRMSEA	ΔSRMR	Decision
Config	31.25(10)	.984	.094	.027					accept

Metrics (loading)	35.25(14)	.984	.079	.040	4.00(4); *p*=.406	.000	.015	.013	accept
Scalar (intercept)	151.22(18)	.899	.175	.094	115.97(4); *p*<.0001	.085	.096	.054	reject

Partial measurement invariance (configural, and metric) was established with all 5 items. Dropping items did not improve the indices; the best indices when dropping items were with 4 items (1-3, 5).

Table S17: Measurement invariance tests for SWLS with 4 items.

Model	χ^2 (df)	CFI	RMSEA	SRMR	$\Delta\chi2$ (Δdf)	ΔCFI	ΔRMSEA	ΔSRMR	Decision
Config	12.88(4)	.991	.096	.017					accept
Metrics (loading)	13.47(7)	.994	.062	.019	.59(3); *p*=.900	.002	.034	.002	reject
Scalar (intercept)	50.69(10)	.961	.130	.059	37.22(3); *p*<.0001	.033	.068	.040	reject

I. State Optimism (SOM-7)

Table S18: Measurement invariance tests for SOM-7 with all 7 items.

Model	χ^2 (df)	CFI	RMSEA	SRMR	$\Delta\chi2$(Δdf)	ΔCFI	ΔRMSEA	ΔSRMR	Decision
Config	29.42(28)	.999	.015	.019					accept
Metrics (loading)	41.50(34)	.996	.030	.047	12.08(6); p=.060	.003	.016	.029	accept
Scalar (intercept)	96.10(40)	.970	.076	.062	54.60(6); p<.0001	.026	.046	.015	reject

Partial measurement invariance (configural, metric, and scalar) was established with 6 of the 7 items (1-6).

Table S19: Measurement invariance tests for SOM-7 with 6 items.

Model	χ^2 (df)	CFI	RMSEA	SRMR	$\Delta\chi2$(Δdf)	ΔCFI	ΔRMSEA	ΔSRMR	Decision
Config	21.94(18)	.997	.030	.018					accept
Metrics (loading)	33.17(23)	.993	.043	.049	11.23(5); *p*=.047	.004	.013	.031	accept
Scalar (intercept)	41.63(28)	.991	.045	.052	8.46(5); *p*=.133	.002	.002	.003	accept

J. Resilience (BRC)

Table S20: Measurement invariance tests for BRC with all 4 items.

Model	χ^2 (df)	CFI	RMSEA	SRMR	Δχ2 (Δdf)	ΔCFI	ΔRMSEA	ΔSRMR	Decision
Config	6.22(4)	.992	.048	.020					accept
Metrics (loading)	11.78(7)	.983	.053	.041	5.57(3); *p*=.134	.009	.005	.022	accept
Scalar (intercept)	97.37(10)	.691	.191	.119	85.57(3); *p*<.001	.292	.137	.078	reject

The models with the best indices after dropping one or more items had 3 items (with items 1-3). Like the full scale, it established metrics invariance, but not scalar invariance.

Table S21: Measurement invariance tests for BRC with 3 items.

Model	χ^2 (df)	CFI	RMSEA	SRMR	Δχ2 (Δdf)	ΔCFI	ΔRMSEA	ΔSRMR	Decision
Config	0 (0)	1.000	.000	.000					accept
Metrics (loading)	1.69(2)	1.000	.000	.020	1.69(2); *p*=.430	.000	.000	.020	accept
Scalar (intercept)	13.88(4)	.954	.101	.049	12.19(2); *p*=.002	.046	.101	.029	reject

K. Meaning in life

Table S22: Measurement invariance tests for MIL with all 10 items.

Model	χ^2 (df)	CFI	RMSEA	SRMR	Δχ2 (Δdf)	ΔCFI	ΔRMSEA	ΔSRMR	Decision
Config	143.84(68)	.971	.068	.047					accept
Metrics (loading)	158.91(76)	.968	.067	.054	15.07 (8); *p*=.058	.003	.001	.007	accept
Scalar (intercept)	233.67 (84)	.943	.086	.065	74.76 (8); *p*<.0001	.026	.019	.011	reject

Partial measurement invariance (configural, metric, and scalar) was established with 8 of the 10 items (1-4,6-8, 10).

Table S23: Measurement invariance tests for MIL with 8 items.

Model	χ^2 (df)	CFI	RMSEA	SRMR	Δχ2 (Δdf)	ΔCFI	ΔRMSEA	ΔSRMR	Decision
Config	98.16(38)	.971	.081	.047					accept
Metrics (loading)	110.09(44)	.968	.079	.056	11.93 (6), *p*=.064	.003	.002	.008	accept
Scalar (intercept)	119.99(50)	.966	.076	.057	9.90(6); *p*=.129	.002	.003	.001	accept

3. Cultural Differences based on measurement invariant items

Only state well-being (WHO-10), state optimism (SOM_7), and meaning in life (MIL-presence, MIL-search) measures established strong (scalar) invariance across the two cultural groups. Thus cultural comparisons can be made on these measures only. The other measures are included in the tables for completeness.

Table S24. Cultural comparisons based on measurement invariance items (Time 1)

		Canada			China					
Measure	Number of items (specific item)	Internal consistency α	Mean	SD	Internal consistency α	Mean	SD	F (dfs)	p	η_p^2
WHO-10	5(1,3,4,6,10)	.84	2.95	1.31	.79	3.83	1.06	75.72 (1,557)	<.001	.12
SWLS	5 (full scale)	.87	4.70	1.26	.87	3.98	1.13			
SOM_7	6 (1-6)	.88	3.30	.83	.84	3.53	.69	13.47(1,556)	<.001	.02
BRC	4 (full scale)	.59	3.73	.59	.69	3.56	.55			
MIL: presence	4 (1,4-6)	.89	4.27	1.34	.84	4.87	.93	36.90 (1,553)	<.001	.06
MIL: search	4 (3,7,8,10)	.88	4.99	1.22	.83	5.16	.87	3.59(1,553)	=.059	.01
LOT-R	6 (full scale)	.83	3.25	.74	.62	3.68	.50			

Table S25. Cultural comparisons based on measurement invariance items (Time 2)

		Canada			China					
Measure	Number of items (specific item)	Internal consistency α	Mean	SD	Internal consistency α	Mean	SD	F (dfs)	p	η_p^2
WHO-10	5(1,3,4,6,10)	.82	3.50	1.34	.79	4.43	1.05	72.36 (1,480)	<.001	.13
SWLS	5	.87	4.57	1.20	.89	4.09	1.13			
SOM_7	6 (1-6)	.90	3.29	.84	.87	3.52	.78	11.07(1,480)	=.001	.02
BRC	4	.66	3.65	.64	.67	3.57	.54			
MIL: presence	3 (1,4,6)	.88	4.12	1.34	.81	4.75	.97	34.20 (1,479)	<.001	.07
MIL: search	5 (2,3,7,8,10)	.92	4.91	1.22	.87	5.09	.84	3.52 (1,479)	=.061	.01

4. Factor loadings based on data from Time 1

Table S26. Factor loadings for Adapted WHO (full scale) at Time 1.

	Canada	China
Item 1	-.58	-.36
Item 2	.74	.49
Item 3	.79	.76
Item 4	.66	.64
Item 5	.79	.79
Item 6	.76	.80
Item 7	.76	.72
Item 8	.73	.40
Item 9	.59	.54
Item 10	.75	.74

Table S27. Factor loadings for Adapted WHO (5 items) at Time 1.

	Canada	China
Item 1	-.59	-.34
Item 3	.80	.83
Item 4	.68	.67
Item 6	.74	.75
Item 10	.74	.72

Table S28. Factor loadings for SWLS (full scale) at Time 1.

	Canada	China
Item 1	.84	.87
Item 2	.73	.81
Item 3	.86	.82
Item 4	.75	.78
Item 5	.64	.50

Table S29. Factor loadings for State Optimism (full scale) at Time 1.

	Canada	China
Item 1	.75	.71
Item 2	.79	.74
Item 3	.86	.78
Item 4	.57	.63
Item 5	.84	.80

Item 6	.70	.52
Item 7	.84	.78

Table S30. Factor loadings for State Optimism (6 items) at Time 1.

	Canada	China
Item 1	.77	.73
Item 2	.78	.73
Item 3	.85	.78
Item 4	.58	.62
Item 5	.85	.79
Item 6	.68	.52

Table S31. Factor loadings for Brief Resilience Coping Scale (full scale) at Time 1

	Canada	China
Item 1	.65	.55
Item 2	.40	.53
Item 3	.71	.71
Item 4	.37	.64

Table S32. Factor loadings for Meaning in Life Scale (full scale) at Time 1

	Canada		China	
	Search	Presence	Search	Presence
Item 1	-.02	.75	.05	.75
Item 2	.75	-.01	.62	.18
Item 3	.79	.19	.69	.10
Item 4	.04	.85	-.03	.87
Item 5	.02	.79	.03	.63
Item 6	-.01	.86	.00	.73
Item 7	.79	.08	.65	-.06
Item 8	.86	-.05	.84	-.02
Item 9	.06	-.65	.03	-.64
Item 10	.79	-.18	.79	-.08

Table S33. Factor loadings for Meaning in Life Scale (8 items) at Time 1

	Canada		China	
	Search	Presence	Search	Presence
Item 1	.02	.76	.04	.76
Item 3	.74	.19	.72	.11
Item 4	.03	.85	-.03	.83
Item 5	.00	.79	.02	.65
Item 6	-.02	.86	-.01	.75
Item 7	.79	.08	.60	-.03
Item 8	.90	-.04	.84	.00
Item 10	.80	-.16	.80	-.05

5. Factor loadings based on data from Time 2

Table S34. Factor loadings for Adapted WHO (full scale) at Time 2.

	Canada	China
Item 1	-.59	-.38
Item 2	.75	.63
Item 3	.71	.75
Item 4	.71	.72
Item 5	.82	.80
Item 6	.75	.74
Item 7	.74	.72
Item 8	.77	.42
Item 9	.65	.58
Item 10	.68	.77

Table S35. Factor loadings for Adapted WHO (5 items) at Time 2.

	Canada	China
Item 1	-.57	-.37
Item 3	.77	.79
Item 4	.74	.71
Item 6	.71	.72
Item 10	.66	.78

Table S36. Factor loadings for SWLS (full scale) at Time 2.

	Canada	China
Item 1	.83	.87

Item 2	.80	.86
Item 3	.89	.87
Item 4	.68	.73
Item 5	.59	.62

Table S37. Factor loadings for State Optimism (full scale) at Time 2.

	Canada	China
Item 1	.81	.71
Item 2	.85	.73
Item 3	.85	.80
Item 4	.59	.72
Item 5	.87	.84
Item 6	.69	.59
Item 7	.81	.69

Table S38. Factor loadings for State Optimism (6 items) at Time 2.

	Canada	China
Item 1	.81	.71
Item 2	.85	.72
Item 3	.85	.81
Item 4	.59	.73
Item 5	.87	.83
Item 6	.69	.60

Table S39. Factor loadings for Brief Resilience Coping Scale (full scale) at Time 2.

	Canada	China
Item 1	.63	.53
Item 2	.63	.60
Item 3	.75	.66
Item 4	.34	.55

Table S40. Factor loadings for Meaning in Life (full scale) at Time 2.

	Canada		China	
	Search	Presence	Search	Presence
Item 1	-.03	.84	.09	.75
Item 2	.80	-.06	.83	.03
Item 3	.79	.16	.76	-.08

Item 4	.03	.87	.01	.84
Item 5	.00	.74	.01	.72
Item 6	.00	.79	-.06	.73
Item 7	.80	.04	.79	-.02
Item 8	.90	.01	.68	.08
Item 9	-.01	-.69	.08	-.63
Item 10	.86	-.11	.74	.01

Table S41. Factor loadings for Meaning in Life (8 items) at Time 2.

	Canada		China	
	Search	Presence	Search	Presence
Item 1	-.03	.86	.08	.74
Item 2	.81	-.07	.82	.05
Item 3	.79	.17	.76	-.07
Item 4	.03	.85	.00	.83
Item 6	.00	.81	-.07	.74
Item 7	.80	.03	.79	-.04
Item 8	.90	.01	.68	.10
Item 10	.86	-.11	.75	-.01

6. **Mediation analyses based on cross-sectional data at Time 1.**
IV = culture (China = .5, Canada = -.5). The tests were done with Hayes' Process 3.5 for SPSS (2018) with 10,000 bootstrapping samples.

Table S42. Mediation analysis based on cross-sectional data at Time 1.

DV	Mediator	a path (Culture → mediator)	b path (Mediator → DV)	c' path (IV → DV controlling for mediator)	ab (indirect effect)	95% CI for indirect effect
Well-being	Optimism	*b* = 0.17, *t* = 2.59, *p* = .0099	*b* = 0.98, *t* = 21.62, *p* < .001	*b* = 0.48, *t* = 6.90, *p* < .001	*b* = 0.16	[0.04, 0.29]
Meaning	Optimism	*b* = 0.17, *t* = 2.66, *p* = .008	*b* = 0.80, *t* = 14.78, *p* < .001	*b* = 0.30, *t* = 3.60, *p* < .001	*b* = 0.14	[0.03, 0.21]
Optimism	Well-being	*b* = 0.65, *t* = 6.87, *p* < .001	*b* = 0.47, *t* = 21.62, *p* < .001	*b* = -0.13, *t* = - 2.68, *p* = .008	*b* = 0.30	[0.22, 0.40]
Optimism	Meaning	*b* = 0.44, *t* = 4.48, *p* < .001	*b* = 0.36, *t* = 14.78, *p* < .001	*b* = 0.02, *t* = 0.32, *p* = .753	*b* = 0.16	[0.09, 0.23]

Optimism = state optimism

Well-being = state well-being
Meaning = meaning presence

7. **Mediation analyses based on cross-sectional data at Time 2.**
   IV = culture (China = .5, Canada = -.5). The tests were done with Hayes' Process 3.5 for SPSS (2018) with 10,000 bootstrapping samples.

   Table S43. Mediation analysis based on cross-sectional data at Time 2.

DV	Mediator	a path (Culture → mediator)	b path (Mediator → DV)	c' path (IV → DV controlling for mediator)	ab (indirect effect)	95% CI for indirect effect
Well-being	Optimism	*b* = 0.17, *t* = 2.48, *p* = .014	*b* = 0.97, *t* = 18.14, *p* < .001	*b* = 0.59, *t* = 7.19, *p* < .001	*b* = 0.17	[0.03, 0.30]
Meaning	Optimism	*b* = 0.17, *t* = 2.52, *p* = .012	*b* = 0.77, *t* = 13.80, *p* < .001	*b* = 0.22, *t* = 2.52, *p* =.012	*b* = 0.14	[0.03, 0.24]
Optimism	Well-being	*b* = 0.75, *t* = 7.16, *p* < .001	*b* = 0.42, *t* = 18.14, *p* < .001	*b* = -0.14, *t* = - 2.58, *p* = .010	*b* = 0.32	[0.23, 0.41]
Optimism	Meaning	*b* = 0.35, *t* = 3.50, *p* < .001	*b* = 0.37, *t* = 13.80, *p* < .001	*b* = 0.05, *t* = 0.77, *p* = .445	*b* = 0.13	[0.06, 0.21]

Optimism = state optimism
Well-being = state well-being
Meaning = meaning presence

8. **Longitudinal Effects**
   Table S44. Longitudinal Effects.

	b	*t*	*p*
**DV: Well-being at T2**			
Well-being at T1	.51	9.66	< .001
Culture	.34	3.93	< .001
Optimism at T1	.28	3.80	< .001
Culture X Optimism	-.23	-2.05	.041
Simple slopes for Euro-Canadians	.40	4.35	< .001
Simple slopes for Chinese	.17	1.83	.068
**DV: Well-being at T2**			
Well-being at T1	.60	13.58	< .001
Culture	.28	3.20	.002
Meaning-presence at T1	.13	2.82	.005
Culture X Meaning-presence	.03	.38	.701
**DV: Meaning-presence at T2**			

Meaning-presence at T1	.66	19.13	< .001
Culture	-.03	-.39	.698
Optimism at T1	.16	3.02	.003
Culture X Optimism	-.08	-.92	.356
**DV: Meaning-presence at T2**			
Meaning-presence at T1	.66	19.39	< .001
Culture	-.08	-1.12	.265
Well-being at T1	.12	3.28	.001
Culture X Well-being	-.02	-.36	.719
**DV: Optimism at T2**			
Optimism at T1	.67	15.47	< .001
Culture	-.03	-.62	.534
Well-being at T1	.06	2.06	.040
Culture X Well-being	.03	.63	.528
**DV: Optimism at T2**			
Optimism at T1	.68	18.49	< .001
Culture	-.02	-.41	.679
Meaning-presence at T1	.07	2.77	.006
Culture X Meaning-presence	.03	.74	.458

9. Culture and co-occurrence of positive and negative affect.

Based on previous research, one may expect stronger dialectical thinking among Chinese participants than Euro-Canadian participants. Unfortunately, we were not able to measure it directly due to resource constraints. However, given that we measured both positive and negative affect, we could examine the co-occurrence of positive and negative affect as a proximate indication of dialecticism, which may manifest itself in the experience of positive and negative affect simultaneously (Spencer-Rodgers et al., 2010). Following previous research, we computed the index of co-occurrence of affect in two ways: (1) MIN – the smallest value of all positive and negative affect ratings (Schimmack, 2001), and (2) Residualized MIN (Grossmann et al., 2016). According to Schimmack (2001), the MIN index (i.e., the intensity of the weaker affect in unipolar ratings) represents the extent to which positive and negative affects are mutually exclusive. Thus, higher numbers indicate more mixed emotions, while lower numbers indicate less mixed emotions. Consistent with the literature, Chinese scored higher than Euro-Canadians on both indices, as seen in Table S1. The findings indicate that the co-occurrence of positive and negative affect was stronger among Chinese than among Euro-Canadian participants. These results seem to suggest that dialecticism was stronger among Chinese participants than Euro-Canadian participants based on their affective experiences.

Table S45: The Co-Occurrence of Positive and Negative Affect.

Index	Country	Mean	*SD*	*F*(df)	*p*	η_p^2
MIN (T1)	China	1.79	1.52	*F*(1,557) = 4.56	.033	.01
	Canada	1.54	1.29			
RESMIN (T1)	China	.23	1.07	*F*(1,557) = 27.99	< .001	.05

	Canada	-.21	.93			
MIN (T2)	China	1.96	1.52	$F(1,480) = 8.07$	.005	.02
	Canada	1.59	1.33			
RESMIN (T2)	China	.31	1.02	$F(1,480) = 48.04$	< .001	.09
	Canada	-.31	.95			

Note. T1 = Time 1; T2 = Time 2.
